# Lactate oxidase/catalase-displaying nanoparticles efficiently consume lactate in the tumor microenvironment to effectively suppress tumor growth

**DOI:** 10.1186/s12951-022-01762-6

**Published:** 2023-01-03

**Authors:** Hyukjun Choi, Mirae Yeo, Yujin Kang, Hyo Jeong Kim, Seong Guk Park, Eunjung Jang, Sung Ho Park, Eunhee Kim, Sebyung Kang

**Affiliations:** Department of Biological Sciences, Ulsan National Institute of Science and Technology (UNIST), Ulsan, 44919 South Korea

**Keywords:** Lactate oxidase, Catalase, Tumor microenvironment, Lactate consumption, Tumor suppression

## Abstract

The aggressive proliferation of tumor cells often requires increased glucose uptake and excessive anaerobic glycolysis, leading to the massive production and secretion of lactate to form a unique tumor microenvironment (TME). Therefore, regulating appropriate lactate levels in the TME would be a promising approach to control tumor cell proliferation and immune suppression. To effectively consume lactate in the TME, lactate oxidase (LOX) and catalase (CAT) were displayed onto *Aquifex aeolicus* lumazine synthase protein nanoparticles (AaLS) to form either AaLS/LOX or AaLS/LOX/CAT. These complexes successfully consumed lactate produced by CT26 murine colon carcinoma cells under both normoxic and hypoxic conditions. Specifically, AaLS/LOX generated a large amount of H_2_O_2_ with complete lactate consumption to induce drastic necrotic cell death regardless of culture condition. However, AaLS/LOX/CAT generated residual H_2_O_2_, leading to necrotic cell death only under hypoxic condition similar to the TME. While the local administration of AaLS/LOX to the tumor site resulted in mice death, that of AaLS/LOX/CAT significantly suppressed tumor growth without any severe side effects. AaLS/LOX/CAT effectively consumed lactate to produce adequate amounts of H_2_O_2_ which sufficiently suppress tumor growth and adequately modulate the TME, transforming environments that are favorable to tumor suppressive neutrophils but adverse to tumor-supportive tumor-associated macrophages. Collectively, these findings showed that the modular functionalization of protein nanoparticles with multiple metabolic enzymes may offer the opportunity to develop new enzyme complex-based therapeutic tools that can modulate the TME by controlling cancer metabolism.

**Graphical Abstract:**

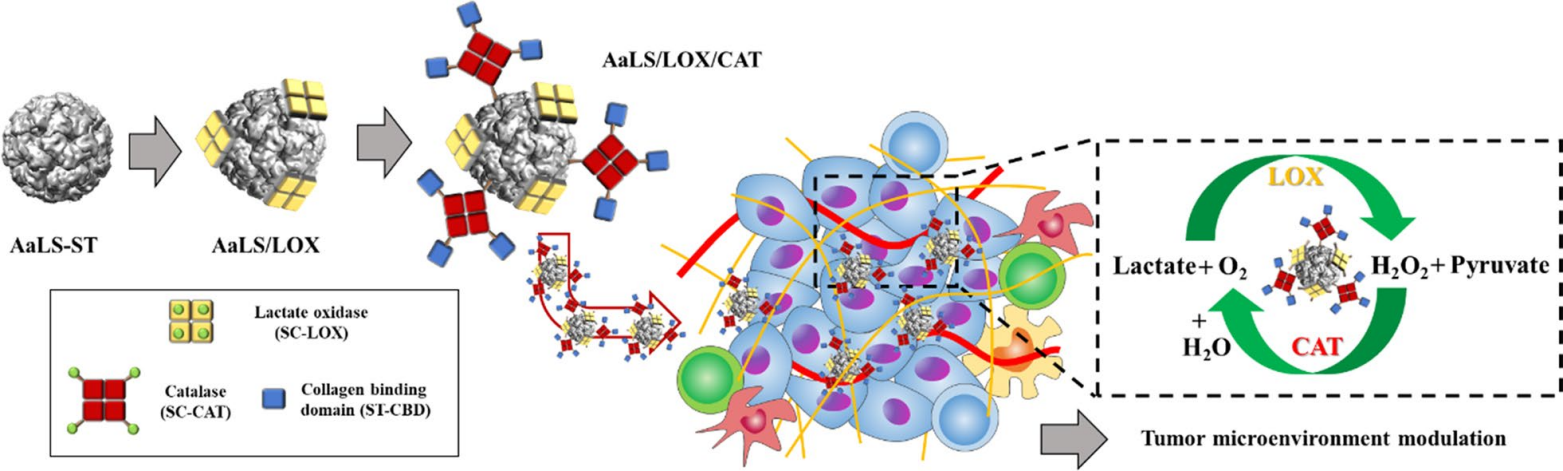

**Supplementary Information:**

The online version contains supplementary material available at 10.1186/s12951-022-01762-6.

## Background

The establishment of tumor masses and its continuous growth induce abnormal angiogenesis and metabolic processes, creating a unique microenvironment that is quite different from normal tissue environments [1]. A tumor microenvironment (TME) refers to the environment around a tumor, including the extracellular matrix (ECM), surrounding immune cells, blood vessels, fibroblasts, signaling molecules, and tumor metabolites [2]. As a result of excessive anaerobic glycolytic processes, collectively known as the Warburg effect, tumor cells exhibit increased glucose uptake that favors their aggressive proliferation, producing large amounts of lactate as a waste product [3]. Therefore, to avoid intracellular acidification, tumor cells rapidly export lactate via monocarboxylate transporters (MCTs), resulting in the accumulation of high lactate concentrations in the TME [4]. Recent studies have shown that a high lactate concentration is not only a metabolic waste product, accounting for the TME acidic condition, but also involves in important metabolic pathways and immune modulations in the TME [5–7]. Moreover, lactate can be used as an energy source when glucose is limited and a carbon source for lipid synthesis in cancer cells [8–10]. Additionally, lactate is an important signaling molecule that interacts with the G-protein coupled receptor subfamily, GPR81 [11]. As previously reported, the autocrine interaction of lactate with GPR81 in tumors promote tumor growth [12], metastasis [13], angiogenesis [14], DNA repair [15], and immune evasion[16]. Besides, the paracrine interaction of lactate with GPR81 in immune cells favors immunosuppressive phenotypes, such as inhibiting CD8^+^ T and natural killer (NK) cells [17, 18], the differentiation of tolerogenic dendritic cells [15], macrophage polarization toward the M2-like phenotype [19], and activating tumor-supportive regulatory T cells [20].

Therefore, since lactate is a crucial molecule in tumor cell proliferation and immune suppression, it has been a promising target of various tumor therapies for TME modulation and tumor rejection. Literature has also reported that targeting lactate metabolism by reducing lactate production through the inhibition of lactate dehydrogenase (LDHA) or promoting lactate secretion through the inhibition of MCTs showed anti-tumor effects [21, 22]. However, although these strategies showed tolerable minor side effects, they are challenging for clinical applications due to their unexpected low drug efficacy in vivo, which is probably because the drugs only reduce the additional lactate production in tumors and cannot remove the high concentration of lactate already present. Hence, to develop better tumor therapy alternatives, there is a massive demand for more effective strategies to remove lactate from the TME.

Recently, lactate oxidase (LOX), which consumes lactate and O_2_ as substrates to produce pyruvate and H_2_O_2_, has shown therapeutic potential by reducing tumor lactate and inducing TME modulation [23–25]. These approaches used nano-sized carriers for LOX delivery to the tumor site, improving the immunosuppressive function of TMEs by activating immune systems in tumor sites. Combination therapy with supplementary drugs, such as immune checkpoint inhibitors and/or small molecule drugs, have also exhibited significant anti-tumor efficacy [23, 25–27]. However, the hypoxic conditions in the TME provide only a limited oxygen supply, leading to reduced LOX activity and lactate consumption in tumor masses [28]. Furthermore, H_2_O_2_ production in normal tissue can result in serious side effects on tissues and organs to be damaged [26]. Hence, this process should increase oxygen supply to the tumor, favoring increased LOX efficacy and decreased cell cytotoxicity to the normal tissues [29–31].

**Fig. 1:**
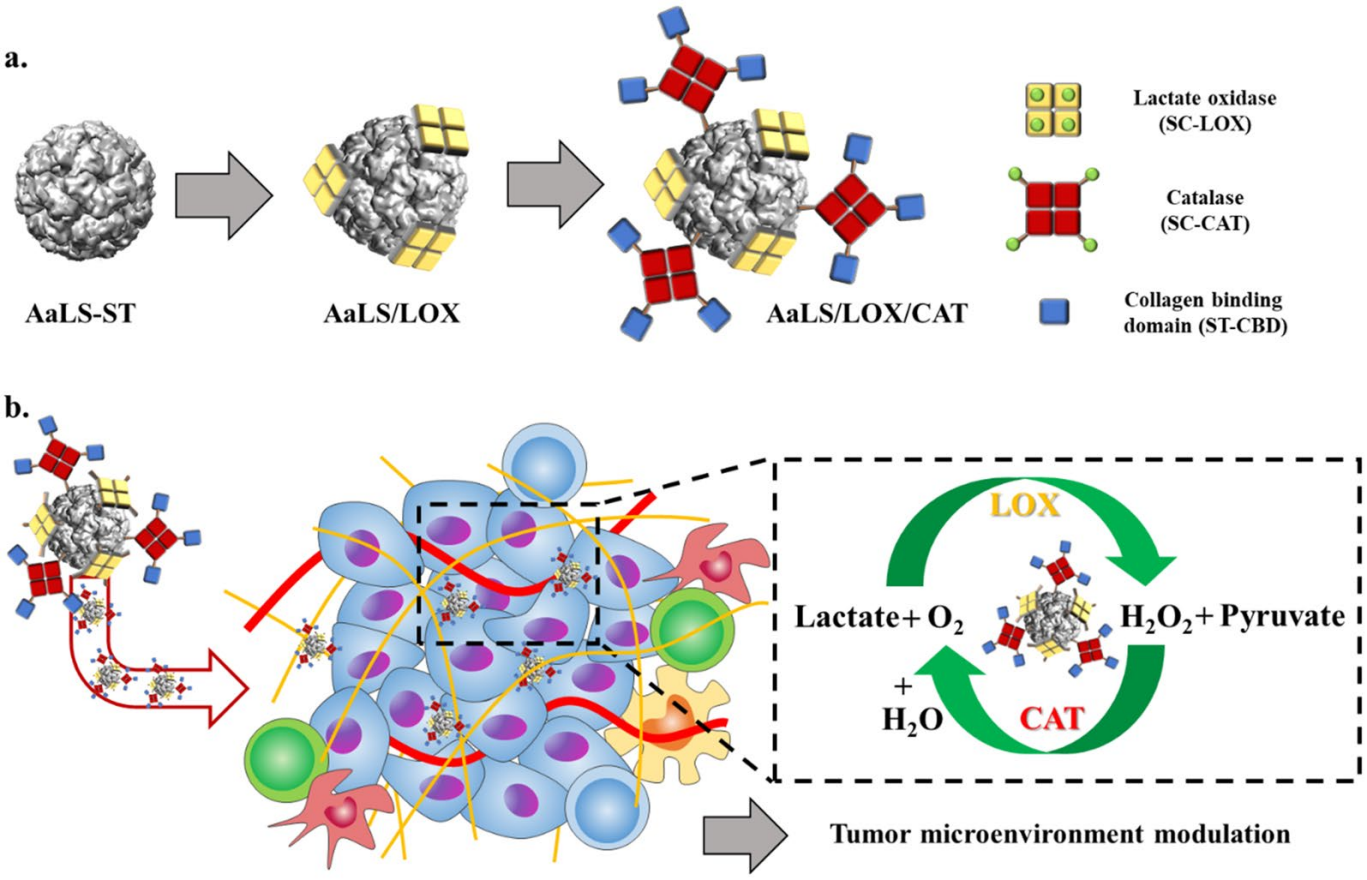
Schematic illustration of constructing AaLS/LOX and AaLS/LOX/CAT and their application to the TME modulation. **a** Polyvalent immobilization of LOX onto the surface of AaLS and subsequent CAT immobilization using a SpyTag/SpyCatcher ligation system. **b** Schematic illustration of AaLS/LOX/CAT delivery to the tumor sites and their subsequent TME modulation by the effective consumption of lactate and H_2_O_2_

Thus, we, here, individually or simultaneously displayed potential therapeutic enzymes, LOX and catalase (CAT), onto previously established *Aquifex aeolicus* lumazine synthase protein nanoparticles (AaLS) [32–34] through a SpyTag/SpyCatcher (ST/SC) bacterial glue system [35], after which we used them to effectively consume lactate and modulate the TME for tumor growth suppression (Fig. 1). Subsequently, the in vivo tumor suppression efficacy of LOX and CAT dual enzyme-displaying AaLS was evaluated using CT26 tumor-bearing mice. Then, the tumor-infiltrated immune cell profiles were investigated to evaluate the impact of lactate consumption in the TME on the alteration of immune cell population. The approach described here would provide opportunities for developing new enzyme complex-based therapeutic tools to modulate the TME by controlling cancer metabolism.

## Results and discussion

### Construction of enzyme-displaying protein nanoparticles through LOX or LOX and CAT ligation on a protein nanoparticle

To develop multiple enzyme-displaying protein nanoparticles, we utilized our previously established AaLS-ST comprising 60 identical subunits as a template [32, 33]. AaLS subunits self-assemble to form an icosahedral structure having outer and inner diameters of 16 and 9 nm, respectively [36]. They also effectively displayed various functional proteins through a ST/SC ligation system [32, 33]. SC was genetically fused to the N-termini of both LOX and CAT to form SC-LOX and SC-CAT, respectively. As a result, the fusion proteins were successfully overexpressed and purified (Additional file 1: Figs. S1 and S2a). The subsequent comparison of size exclusion chromatography (SEC) elution profiles with molecular weight standards revealed that both SC-LOX and SC-CAT maintained their tetrameric configuration (Additional file 1: Fig. S2b, c). In addition, SC fusion proteins were eluted slightly earlier than wild-type proteins, indicating a size increase due to the SC fusion (Additional file 1: Fig. S2b). Therefore, we conducted band intensity analyses of sodium dodecyl sulfate–polyacrylamide gel electrophoresis (SDS-PAGE) of individual proteins and ligated forms to determine and control the relative amount of displayed proteins on a AaLS, using the NIH Image J, which is one commonly used analytical technique to determine the relative band intensities of multiple components in SDS-PAGE [37, 38]. Results from ST/SC ligations between AaLS-ST and either SC-LOX or SC-CAT showed that while a successful covalent connection with both proteins exhibited new bands around 100 kDa in SDS-PAGE (Additional file 1: Fig. S2d, e), spontaneous protein aggregation was induced by an ST/SC ligation between AaLS-ST and SC-CAT (AaLS/CAT). Although approximately 40% of ligated proteins were precipitated upon ligation between AaLS-ST and SC-CAT and removed by centrifugation, no apparent precipitation was observed upon ST/SC ligation between AaLS-ST and SC-LOX (AaLS/LOX) (Additional file 1: Fig. S2d, e). Structural analyses based on the crystal structures of LOX and homologous CAT suggested that the LOX tetramer had a C4 symmetry, causing all N-termini of LOX subunits to face the same direction (Additional file 1: Fig. S3a, red-colored spheres) [39] and the CAT tetramer was tetrahedrally arranged with two oppositely facing N-termini of four CAT subunits (Additional file 1: Fig. S3b, blue-colored spheres) [40]. Thus, multiple SC-LOXs could interact with only one AaLS-ST nanoparticle, decorating the surface without forming further clusters with nascent nanoparticles (Fig. 1a), whereas one SC-CAT was able to interact with two or more AaLS-ST particles to form a large interparticle network, leading to protein aggregation and subsequent precipitation. Therefore, SC-CAT tetramers were first partially pre-blocked with the ST-fused small collagen-binding domain (ST-CBD) afterward to prevent interparticle aggregation, and subsequently ligated to AaLS-ST (Additional file 1: Fig. S2f) and SDS-PAGE analyses of prior reactions between ST-CBD and SC-CAT, and the subsequent reaction with AaLS-ST, revealed that the partial passivation of SC-CAT with ST-CBD rescued protein aggregation and precipitation (Additional file 1: Fig. S2g, h).

**Fig. 2:**
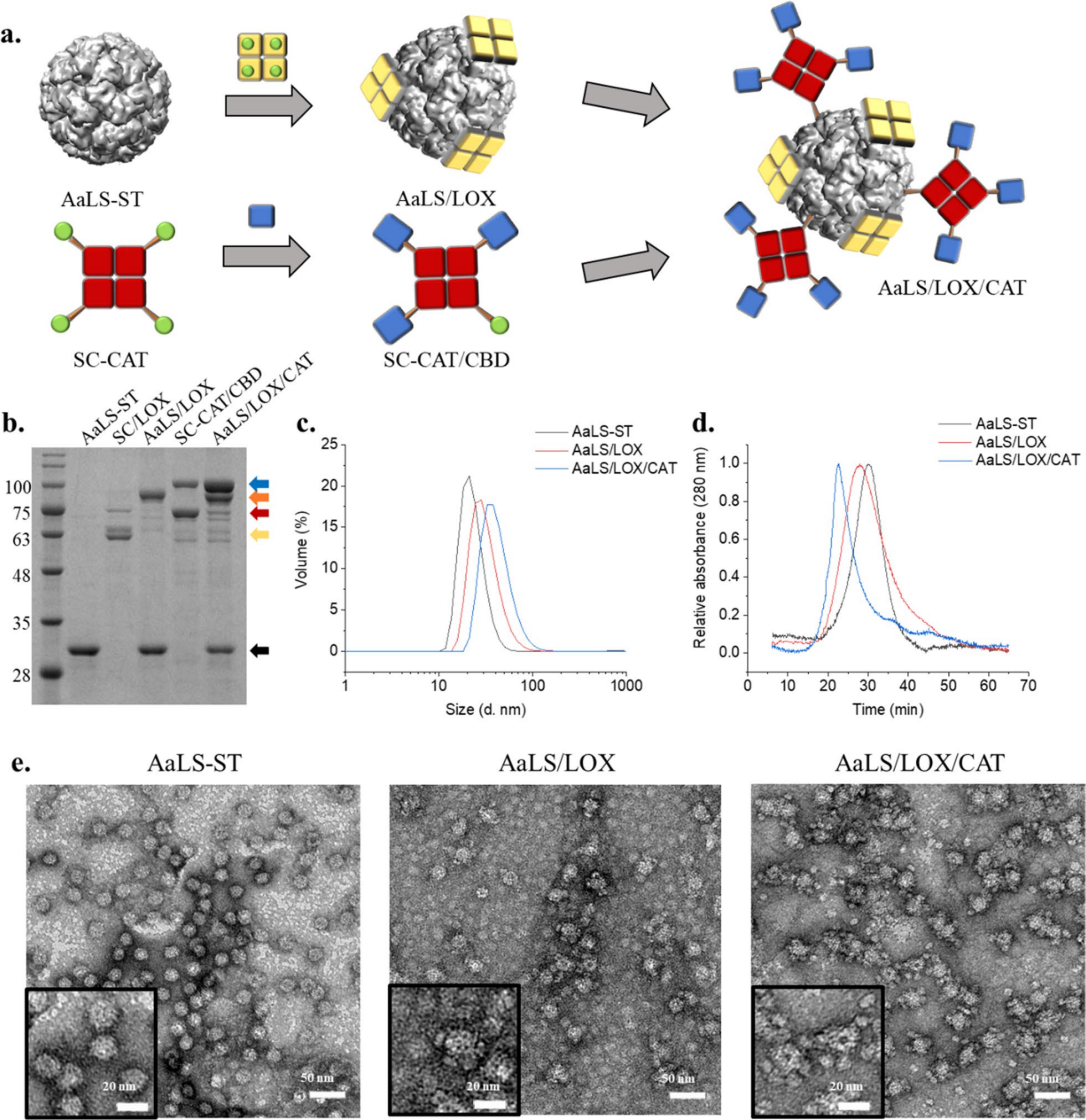
Construction and characterization of AaLS/LOX and AaLS/LOX/CAT. **a** Schematic illustration of AaLS/LOX and AaLS/LOX/CAT construction. **b** SDS-PAGE analyses of the individual proteins and their reaction resultants. **c** DLS analyses of AaLS-ST (22.9 ± 6.6 nm; PDI, 0.296), AaLS/LOX (31.5 ± 11.4 nm; PDI, 0.235), and AaLS/LOX/CAT (41.9 ± 15.4 nm; PDI, 0.139). **d** SEC elution profiles of AaLS-ST, AaLS/LOX, and AaLS/LOX/CAT. **e** TEM images of AaLS-ST, AaLS/LOX, and AaLS/LOX/CAT, stained with 1% uranyl acetate

Subsequently, LOX-displaying protein nanoparticles were generated through the ligation of SC-LOX with AaLS-ST to form AaLS/LOX. However, LOX-and-CAT-dual enzyme-displaying protein nanoparticles were formed through the ligation of CBD-passivated SC-CAT (SC-CAT from here for simplicity) to AaLS/LOX to form AaLS/LOX/CAT (Fig. 2a). Equivalent amounts of CBD-passivated SC-CAT and SC-LOX were ligated to AaLS-ST and approximately 20% of AaLS-ST subunits remained unmodified (Fig. 2b, black arrow). Nevertheless, the ligation reactions between AaLS-ST and SC-LOX or AaLS-ST and SC-CAT, revealed new bands of approximately 85 or 100 kDa in the SDS-PAGE which were well matched with a molecular weight of the ligated AaLS-ST and SC-LOX subunits (84.6 kDa, Fig. 2b, orange arrow) and those of the ligated SC-CAT and AaLS-ST subunits (95.8 kDa) or SC-CAT and ST-CBD subunits (98.8 kDa) (Fig. 2b, blue arrow). Nonetheless, the molecular weights of ST-CBD (26.2 kDa) and AaLS-ST subunits (29.2 kDa) were similar to each other (Fig. 2b, black arrow), and the ligated products with SC-CAT subunits exhibited similar molecular weights (Fig. 2b, blue arrow). SC-LOX ligation and subsequent SC-CAT ligation onto the AaLS-ST resulted in increased hydrodynamic diameters (22.9, 31.5, 41.9 nm, respectively) in dynamic light scattering (DLS) analyses and an earlier elution in SEC, suggesting that the particle size increased upon enzyme immobilization (Figs. 2c, d), as illustrated in Fig. 2a. Additionally, transmission electron microscopic (TEM) images showed stable spherical nanoparticle architectures in each protein and both AaLS/LOX and AaLS/LOX/CAT exhibited extra-electron densities around the nanoparticles (Fig. 2e), as illustrated in Fig. 2a. Notably, however, the TEM images of both LOX and CAT showed smaller particles of approximately 5 nm (Additional file 1: Fig. S3c), similar to the extra densities observed in AaLS/LOX and AaLS/LOX/CAT (Fig. 2e and insets). The zeta potential values of AaLS-ST, AaLS/LOX, and AaLS/LOX/CAT were − 13.6 mV, − 13.2 mV, and − 14.9 mV, respectively, (Additional file 1: Fig. S2j) and there was no significant difference among them observed. Therefore, these results collectively indicate that both LOX and CAT simultaneously and successfully ligated and displayed onto the surface of the AaLS-ST to form dual-enzyme-displaying nanoparticles.

### AaLS/LOX and AaLS/LOX/CAT efficiently consumed lactate in the lactate-containing buffer

**Fig. 3:**
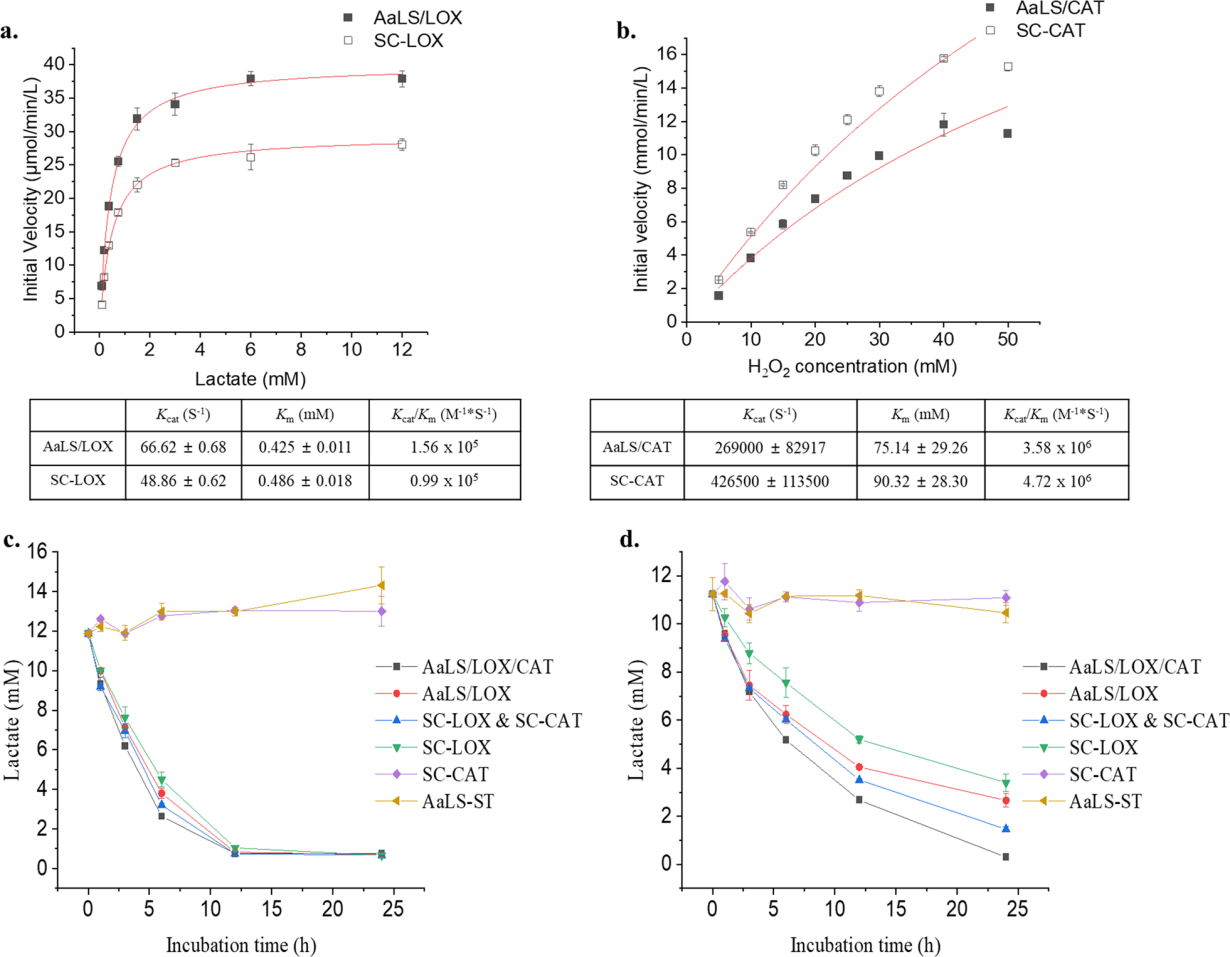
Catalytic activities of LOX and CAT variants and their lactate consumption profiles under either normoxic or hypoxic conditions. Initial velocity plots showing LOX (**a**) and CAT (**b**) variants and their Michaelis–Menten enzymatic kinetic parameters. Lactate consumption profiles in the buffer under normoxic (**c**) and hypoxic (**d**) conditions are also shown

We also evaluated the enzymatic activities of LOX and CAT variants. First, we prepared and measured each enzyme’s typical Michaelis–Menten kinetic parameters. Then, the enzymatic activities of LOX variants (SC-LOX and AaLS/LOX) were evaluated by monitoring the amounts of produced H_2_O_2_ that underwent subsequent reactions with Amplex Red, using horseradish peroxidase (HRP) to develop colorimetric signals [41]. On the other hand, the enzymatic activities of CAT variants (SC-CAT and AaLS/CAT) were determined by measuring ammonium molybdate’s consumption amounts, reacting with residual H_2_O_2_, generating colorimetric signals at 405 nm [42]. Although SC-LOX and AaLS-LOX or SC-CAT and AaLS-CAT exhibited similar enzyme kinetic parameters, no significant enzymatic activity alterations were observed even after the ligation to AaLS-ST (Fig. 3a, b). Furthermore, compared with the reported LOX and CAT kinetic parameters [41, 43], while CAT showed similar enzyme kinetic behavior [43], LOX showed a slightly reduced enzymatic activity, probably due to the N-terminal fusion of SC [41].

Next, we tested the lactate consumption capability of each sample in the buffer. First, 10 mM lactate was added to phosphate-buffered saline (PBS) at pH 6.7 to mimic the TME, after which lactate consumption was monitored under normoxic and hypoxic conditions. Under normoxic condition, all LOX-containing samples (SC-LOX, SC-LOX & SC-CAT, AaLS/LOX, and AaLS/LOX/CAT) rapidly consumed lactate and completed reactions within 12 h, in an almost identical pattern. In contrast, no noticeable lactate consumption was observed in the samples treated with AaLS-ST or SC-CAT (Fig. 3c). Under hypoxic condition, however, LOX-containing samples showed delayed lactate consumption profiles, probably due to the limited oxygen supply (Fig. 3d). We also observed that the samples treated with a combination of LOX and CAT (SC-LOX & SC-CAT and AaLS/LOX/CAT; blue triangles and black squares, respectively) exhibited increased lactate consumption levels compared with those of the LOX-only variants (SC-LOX and AaLS/LOX). These results suggest that co-existing CAT supplies additional oxygen by converting produced H_2_O_2_ to water and oxygen, allowing LOX to more efficiently convert lactate, even during a limited oxygen supply.

### AaLS/LOX induced drastic necrotic cell death but AaLS/LOX/CAT did not alter cell viability with complete lactate consumption

**Fig. 4:**
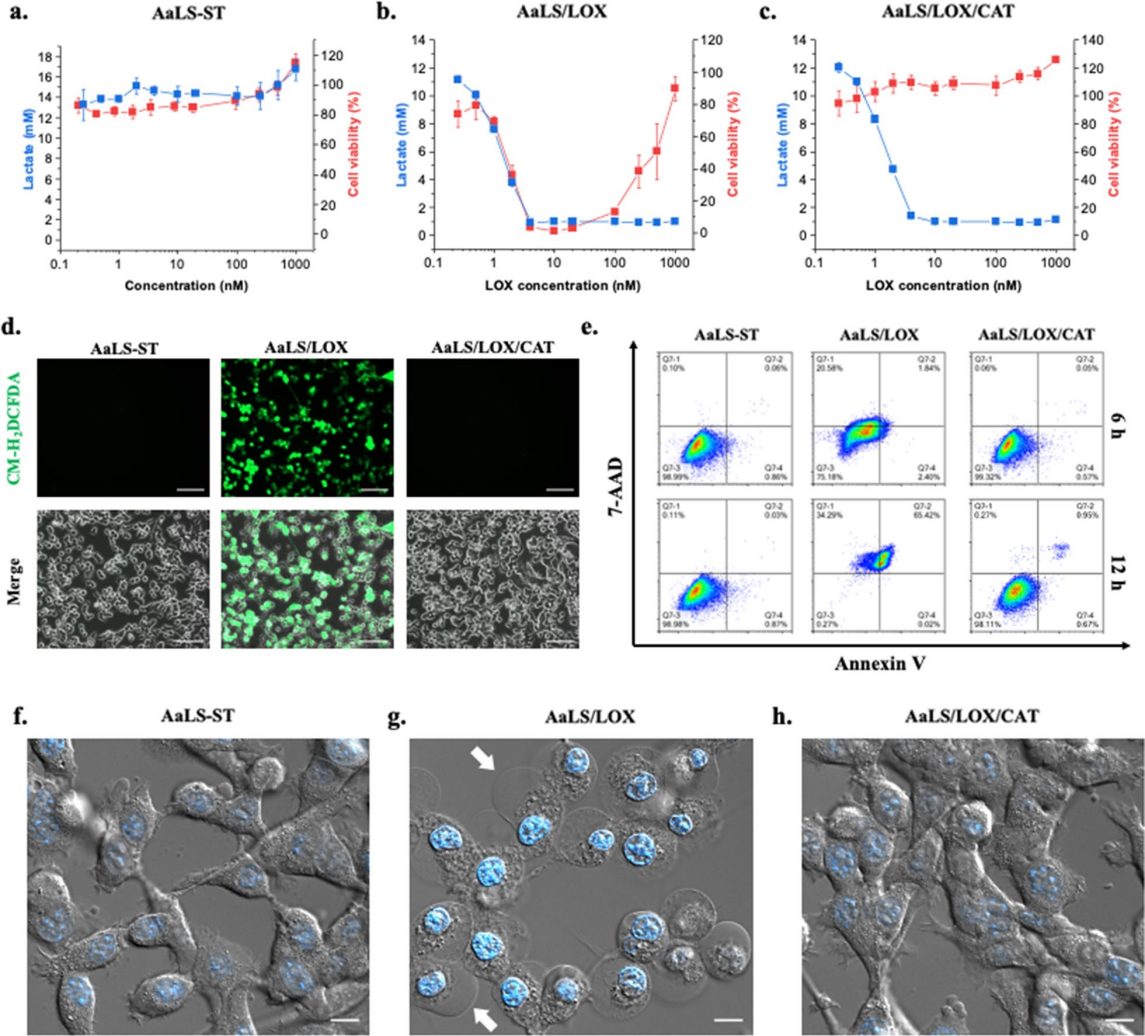
Lactate consumption and cell viability of CT26 cells under normoxic conditions after AaLS-ST, AaLS/LOX, or AaLS/LOX/CAT treatments. The lactate concentration and cell viability profiles of CT26 cells in the media treated with AaLS-ST (**a**), AaLS/LOX (**b**), or AaLS/LOX/CAT (**c**) at various sample concentrations. **d** Fluorescence microscopic images showing CT26 cells treated with AaLS-ST, AaLS/LOX, or AaLS/LOX/CAT, followed by incubation with 2 μM CM-H_2_DCFDA in the dark for 30 min at 37 °C (scale bar, 100 μm). **e** Flow cytometric results showing annexin V/7-AAD-stained CT26 cells upon treatment with AaLS-ST, AaLS/LOX, or AaLS/LOX/CAT. All cells were analyzed after 6 or 12 h of sample treatments. In each plot, the upper-right quadrant indicates late apoptosis and necrosis, the lower-right quadrant indicates early apoptosis, and the lower-left quadrant indicates the normal state of cells. The relative populations of each quadrant were individually marked. Real-time live imaging of CT26 cells treated with AaLS-ST (**f**), AaLS/LOX (**g**), and AaLS/LOX/CAT (**h**) are also shown. The nuclei of cells were stained with Hoechst 33342 and visualized in blue (scale bar, 10 μm)

LOX not only consumes lactate but also generates H_2_O_2_, damaging and eventually killing cells. However, CAT converts highly toxic H_2_O_2_ to oxygen and water, which are beneficial to cells. Therefore, we evaluated the lactate consumption capability and cytotoxic effect of AaLS/LOX and AaLS/LOX/CAT in a cell culture system. First, we prepared a CT26 mouse colorectal carcinoma cell-line and monitored lactate concentrations in the cell culture medium under normoxic conditions at various incubation times (Additional file 1: Fig. S4a). Lactate concentrations gradually increased from 4 mM at 24 h to 12 mM at 48 h, reaching approximately 18 mM after a 72-h culture (Additional file 1: Fig. S4a). Next, CT26 cells were cultured for 48 h to maintain the lactate rich environment followed by treating with samples (AaLS-ST, AaLS/LOX, or AaLS/LOX/CAT) in a concentration-dependent manner. After an additional culturing period of 24 h (72 h total), lactate concentrations and cell viabilities were evaluated (Fig. 4a–c). Although the lactate concentrations of CT26 cells treated with AaLS-ST remained unchanged, even at a high concentration (1 μM) (Fig. 4a, blue squares), almost all lactate produced by CT26 cells were consumed upon treatment with 4 nM or higher concentrations of either AaLS/LOX (Fig. 4b, blue squares) or AaLS/LOX/CAT (Fig. 4c, blue squares). Furthermore, as expected, CT26 cells treated with 4–100 nM of AaLS/LOX were almost completely killed (Fig. 4b, red squares) and their cell morphology was significantly altered (Additional file 1: Fig. S4b). However, 250 nM and higher concentrations of AaLS/LOX allowed the survival of CT26 cells (Fig. 4b, red squares). We hypothesized that the recovered cell viability at 250 nM and higher concentrations of AaLS/LOX-treated cells was due to the explosive conversion of lactate to pyruvate and H_2_O_2_, which subsequently reacted each other to produce acetate, CO_2_, and water, resulting in a rapid reduction of toxic H_2_O_2_ in the culture media [44]. External addition of pyruvate to the culture media indeed completely rescued AaLS/LOX-treated CT26 cells from necrotic cell death (Additional file 1: Fig. S4c), supporting the hypothesis that pyruvate effectively scavenges H_2_O_2_ to protect cells from excessive oxidative stress. We also used a thiol-containing antioxidant, N-Acetyl-L-cysteine (NAC), that can indirectly scavenge H_2_O_2_ by maintaining reduced glutathione levels [45, 46] and observed that NAC partially rescued AaLS/LOX-induced cell death (Additional file 1: Fig. S4c). However, other reactive oxygen species (ROS) scavengers, superoxide dismutase (SOD) mimetics Mn(III) tetrakis (4-benzoic acid) porphyrin chloride (MnTBAP) and Cu(II) diisopropylsalicylate (CuDIPS) did not rescue CT26 cells from AaLS/LOX-induced cell death (Additional file 1: Fig. S4c). Although AaLS/LOX produced significant amounts of H_2_O_2_ leading to necrotic CT26 cell death, AaLS/LOX/CAT did not induce noticeable cell death, even with effective lactate consumption (Fig. 4c). Furthermore, morphological changes were not detected in CT26 cells treated with AaLS/LOX/CAT (Additional file 1: Fig. S4b). Therefore, we concluded that the maintenance of the cell viability of CT26 cells treated with AaLS/LOX/CAT was due to the additional CAT activity, converting toxic H_2_O_2_ to nontoxic oxygen and water, even with the complete consumption of lactate. Fluorescence microscopy using the cell-permeable ROS indicator, 2′,7′-dichlorodihydrofluorescein diacetate (CM-H_2_DCFDA), also showed that while the AaLS/LOX treatment dramatically increased ROS levels, the AaLS/LOX/CAT treatment did not produce ROS in CT26 cells, indicating the effective H_2_O_2_-removing capability of AaLS/LOX/CAT (Fig. 4d).

Subsequently, we investigated the cell death mode induced by AaLS/LOX, using CT26 cells stained with annexin V and 7-amino-actinomycin D (7-AAD) after treatment with AaLS-ST, AaLS/LOX, or AaLS/LOX/CAT. The population of AaLS/LOX-treated CT26 cells began to shift to the 7-AAD positive population after six hours post-sample treatment. Furthermore, they finally shifted to both 7-AAD and annexin V-positive populations at 12 h post-sample treatment, indicating the induction of necrotic cell death (Fig. 4e). However, the population of AaLS/LOX/CAT-treated CT26 cells did not shift to either annexin V or the 7-AAD positive population, similar to that of AaLS-ST-treated CT26 cells (Fig. 4e). Additionally, live cell imaging capture results showed that while AaLS-ST- or AaLS/LOX/CAT-treated CT26 cells maintained their cell morphology and membrane integrity (Figs. 4f, h), AaLS/LOX-treated CT26 cells were extensively swelled, and their membranes were rapidly ruptured (Fig. 4g). These results suggest that although AaLS/LOX treatment produces a large amount of H_2_O_2_ by rapidly consuming the surrounding lactate, inducing drastic necrotic cell death, AaLS/LOX/CAT converts toxic H_2_O_2_ to nontoxic oxygen and water, rescuing cells from ROS-induced cell death even with complete lactate consumption.

### AaLS/LOX and AaLS/LOX/CAT consumed lactate similarly under hypoxic conditions but exhibited different cell cytotoxicity

**Fig. 5:**
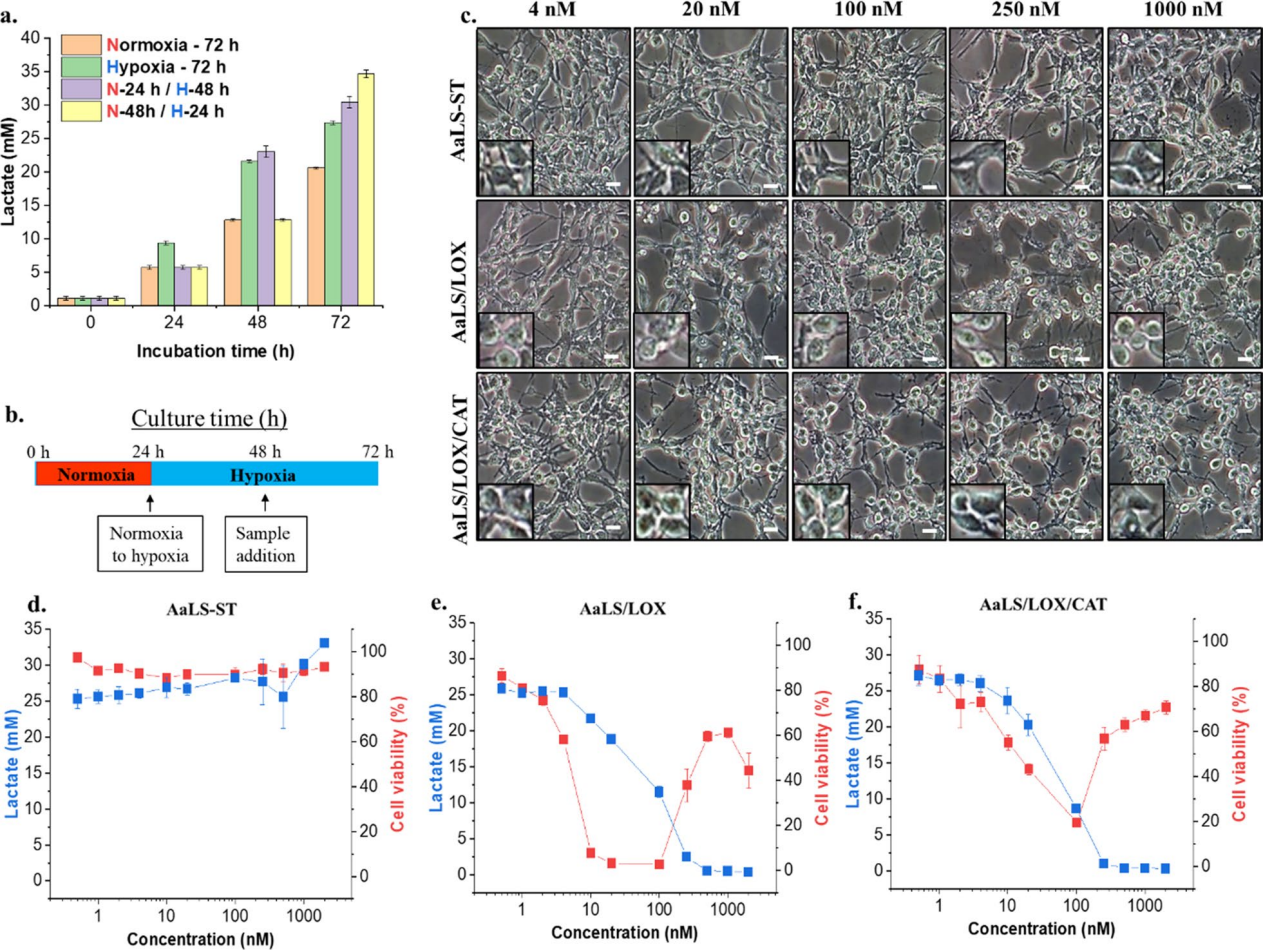
Lactate consumption and cell viability of CT26 cells under hypoxic conditions after AaLS-ST, AaLS/LOX, or AaLS/LOX/CAT treatments. **a** Lactate concentrations at various CT26 cell incubation times under various culture conditions. Normorxia-72 h and Hypoxia-72 h: 72 h culture in normoxic and hypoxic conditions, respectively. N-24 h/H-48 h: 24 h initial culture in normoxia and 48 h following culture in hypoxia. N-48 h/H-24 h: 48 h initial culture in normoxia and 24 h following culture in hypoxia. **b** CT26 cell culture conditions and sample treatment schemes. **c** Microscopic images showing CT26 cells treated with varying amounts of AaLS-ST, AaLS/LOX, or AaLS/LOX/CAT under hypoxic conditions (scale bar, 100 μm). Enlarged individual cell figures are inserted as insets. Lactate concentration and cell viability profiles of CT26 cells in the media treated with AaLS-ST (**d**), AaLS/LOX (**e**), or AaLS/LOX/CAT (**f**) at various sample concentrations are also shown

Since the TME was lactate-rich but oxygen-deficient, the lactate consumption and H_2_O_2_ conversion patterns by LOX and CAT would be different from those under normoxic conditions. Therefore, to mimic the hypoxic TME condition, we cultured CT26 cells under 1% O_2_ condition at different time intervals and monitored their lactate production capabilities (Fig. 5a, green bars). CT26 cells produced more lactate when initially cultured under the normoxic condition and transferred to hypoxic condition (Fig. 5a; purple and yellow bars). Subsequently, we hypothesized that initial tumor formation occurs in vivo under normoxic condition and then the environment could transform to a hypoxic condition as the tumor grows to form a unique TME. Therefore, we initially cultured CT26 cells under a normoxic condition for 24 h and then transferred them to a hypoxic chamber and subjected them to incubation for a further 24 h. After, we treated these cells concentration-dependently with each sample and incubated them for an additional 24 h under the same hypoxic condition (Fig. 5b). Microscopic images of either AaLS/LOX- or AaLS/LOX/CAT-treated CT26 cells showed different cell morphologies from those of AaLS-ST-treated CT26 cells at different concentrations (Fig. 5c). Although AaLS/LOX/CAT and AaLS/LOX consumed lactate from CT26 cell cultures under hypoxic condition (Fig. 5e, f, blue squares), larger LOX amounts were required to consume lactate completely than those under normoxic condition (Fig. 4b, c), suggesting that the limited oxygen supply under hypoxic condition might slow down the LOX activity and more LOX was required to effectively consume lactate. Interestingly, in contrast to normoxic condition, AaLS/LOX/CAT-treated CT26 cells under hypoxic condition showed reduced cell viability throughout whole concentration ranges and the lowest cell viability was observed around 100 nM of AaLS/LOX/CAT (Fig. 5f, red squares). Moreover, fluorescence microscopy using CM-H_2_DCFDA of AaLS-ST, AaLS/LOX, and AaLS/LOX/CAT-treated CT26 cells under the hypoxic condition revealed that while AaLS/LOX/CAT induced weak ROS generation after 12 h treatment, AaLS/LOX markedly increased ROS levels after 4 h treatment, subsequently killing most of the cells (Additional file 1: Fig. S5). The reduced cell viability of AaLS/LOX/CAT treated-CT26 cells at medium concentration (10–100 nM) under hypoxic condition may result from the different enzyme kinetic behavior of LOX and CAT. Both *V*_max_ and *K*_m_ values of CAT were higher than those of the LOX (Fig. 3a, b), suggesting that H_2_O_2_ produced by LOX was incompletely removed by CAT, and this residual H_2_O_2_ sufficiently accumulated enough to damage cells under hypoxic condition, leading to cell death. Collectively, these results imply that AaLS/LOX/CAT can effectively consume lactate to produce small amounts of cytotoxic H_2_O_2_, inducing cell death under the hypoxic TME while reducing its side effects, such as burst production of toxic H_2_O_2_ production, possibly driven by AaLS/LOX to normal cells under normoxic condition.

### Local administration of AaLS/LOX/CAT suppressed tumor growth and altered the TME

**Fig. 6:**
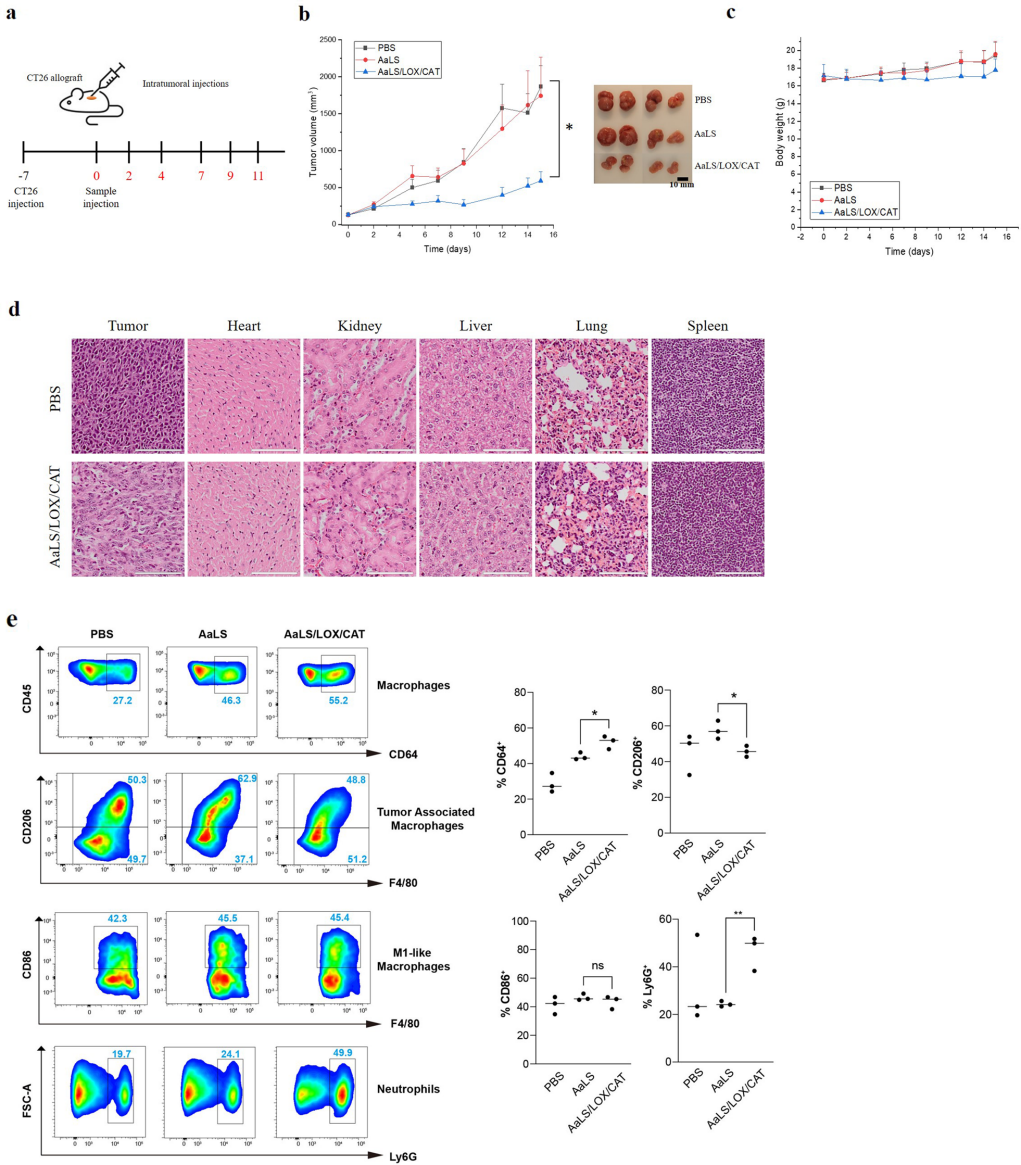
In vivo therapeutic efficacy of AaLS/LOX/CAT. **a** Timeline of the establishment of the CT26 allograft mouse model, the intratumoral injections of samples, and the tumor size measurements. **b** Tumor sizes were measured in mice treated with PBS (black squares), AaLS (red circles), or AaLS/LOX/CAT (blue triangles) every two or three days using a caliper. Data are averages ± standard deviations (SD); n = 4 per group; *p = 0.015. **c** Body weights of mice treated with PBS (black squares), AaLS (red circles), or AaLS/LOX/CAT (blue triangles). **d** Tumors, hearts, kidneys, livers, lungs, and spleens collected from sacrificed mice treated with PBS or AaLS/LOX/CAT were fixed in 10% formalin, embedded in paraffin, and sectioned. Then, the tissue sections were stained with H&E, after which images were captured using an Olympus virtual microscope (scale bar, 100 μm). **e** Representative FACS dot plots showing CD64^+^ macrophages, CD206^+^ or CD86^+^ macrophages gated on CD11b^+^F4/80^+^ cells, and Ly6G^+^ neutrophils gated on CD3^–^CD11b^+^ cells in tumors of indicated conditions. Graph on the right shows the quantification of the frequency of CD64^+^, CD206^+^, CD86^+^ and Ly6G^+^ cells (n = 3). *p < 0.05, **p < 0.01, ns, not significant, by student’s *t*-test

Finally, we explored the translational implications of our findings by examining the efficacy of AaLS/LOX and AaLS/LOX/CAT in CT26 tumor-bearing allografted mice. Since the significant H_2_O_2_ amount generated by AaLS/LOX caused severe necrotic cell death in vitro, we first tested whether AaLS/LOX and AaLS/LOX/CAT can be used in vivo without severe side effects. Various AaLS/LOX or AaLS/LOX/CAT concentrations were locally injected to the tumor site every two or three days for six injections to the CT26 tumor-bearing mice (Fig. 6a). As controls, PBS and AaLS were also administrated based on the same schedule. After the CT26 tumor-bearing mice were treated with either 100 μL of 5 μM or 10 μM AaLS/LOX (molar concentration of LOX subunits) samples, mice begun to die a day after administration (Additional file 1: Fig. S6a; purple and green lines). These results suggest that the significant H_2_O_2_ amount generated by AaLS/LOX caused severe necrotic cell death around the tumor site to kill mice. In contrast, none of the mice died or lost body weight after administration of the same amount of AaLS/LOX/CAT (Additional file 1: Fig. S6a, b; blue and red lines), showing moderate tumor suppressive efficacy (Additional file 1: Fig. S6c). Therefore, we decided not to use AaLS/LOX for in vivo efficacy evaluation any further and increase the AaLS/LOX/CAT doses to enhance tumor suppression efficacy.

We further tested 10 and 20 μM AaLS/LOX/CAT and obtained effective tumor suppression efficacy (Additional file 1: Fig. S6d, e). These AaLS/LOX/CAT concentrations maintained the capability of tumor suppression but did not show serious adverse effects (Additional file 1: Fig. S6d, e). Therefore, we decided to use 10 μM AaLS/LOX/CAT for in vivo efficacy evaluation. CT26 allografted mice were locally injected to the tumor site every two or three days with 100 μL of 10 μM AaLS/LOX/CAT for six injections (Fig. 6a). While AaLS did not affect tumor growth, notably, AaLS/LOX/CAT treatments significantly suppressed tumor growth (Fig. 6b). Additionally, no weight loss and mouse death were observed (Fig. 6c), and there was no noticeable tissue damage in major organs (Fig. 6d). These results suggest that AaLS/LOX/CAT successfully inhibits tumor growth by depleting lactate in the TME without severe side effects.

Numerous publications over the past decades have shown that lactate in the TME modulates tumor cells as well as the immune microenvironment to promote tumor growth [19, 47–52]. Indeed, tumor-derived lactate in the TME can impair the functions of anti-tumor immune cells, including CD8^+^ T and NK cells, supporting the generation of immunosuppressive tumor-associated macrophages (TAMs) [19, 48, 49]. To investigate whether lactate consumption in the TME could impact the alteration of immune cell populations, we collected tumor masses and profiled immune cells from CT26 tumor-bearing mice injected with PBS, AaLS, or AaLS/LOX/CAT. Freshly resected tumors were collected and analyzed by a flow cytometry to define total macrophages, TAMs (M2-like), neutrophils, T cells, and NK cells (Fig. 6e and Additional file 1: Fig. S7a). We observed that while AaLS alone increased CD64^+^ (total) macrophages, AaLS/LOX/CAT further increased infiltrating CD64^+^ macrophages (Fig. 6e, top row). These results suggest that even though protein nanoparticles alone did not affect tumor growth inhibition, they affected the recruitment of macrophage. Additionally, lactate removal further increased macrophage intratumoral intrusion. Of note, we observed that the depletion of lactate by AaLS/LOX/CAT significantly reduced CD206^+^, F4/80^+^ tumor-supportive M2-like TAMs (Fig. 6e, second row), indicating that lactate-induced TAM production was strongly suppressed [51]. In contrast, AaLS moderately increased TAMs associated with poor tumor suppressive efficacy and both AaLS and AaLS/LOX/CAT did not affect M1 activation. Consistently, the depletion of lactate by AaLS/LOX/CAT in mouse bone marrow-derived macrophages significantly reduced the expression of Arg1 and Vegf, which are well-known markers of TAMs (Additional file 1: Fig. S8). Therefore, we proposed that a decrease in TAMs and increased total macrophages, including M0, contributed to the tumor suppressive efficacy. Next, we investigated the proportion of neutrophils (Ly6G^+^) with potent cytotoxic and anti-tumor activities [47]. Interestingly, only AaLS/LOX/CAT highly increased neutrophils (Fig. 6e, bottom row) and these findings indicate that AaLS/LOX/CAT specifically affected the neutrophils’ activity. Furthermore, the proportion of T cells was changed. Treatments with AaLS or AaLS/LOX/CAT increased CD4^+^ T cells and decreased CD8^+^ T cells (Additional file 1: Fig. S7b). Specifically, AaLS/LOX/CAT showed the highest increase in the CD4^+^/CD8^+^ ratio associated with increased immune function. However, the proportion of NK cells was insignificantly affected (Additional file 1: Fig. S7b). Overall, our findings suggest that the specific clearance of lactate by AaLS/LOX/CAT altered the immune environment in favor of anticancer functions, such as neutrophil increase and TAMs decrease, showing clear translational implications for tumor therapy.

## Conclusions

In this study, we prepared AaLS as enzyme-displayable nanoplatforms and displayed potential therapeutic enzymes, LOX or LOX and CAT, onto the surface of AaLS through a SpyTag/SpyCatcher (ST/SC) bacterial glue system to form AaLS/LOX and AaLS/LOX/CAT. Successful immobilizations of LOX or LOX and CAT onto the surface of AaLS without any noticeable nanoparticle architectural alteration were confirmed by TEM, DLS, and SEC analyses.

Although both AaLS/LOX and AaLS/LOX/CAT efficiently consumed lactate in the buffer under normoxic and hypoxic conditions, they exhibited delayed lactate consumption under the hypoxic condition, probably due to the limited oxygen supply. However, AaLS/LOX/CAT showed increased levels of lactate consumption compared with AaLS/LOX, probably owing to the additional oxygen produced by CAT.

In a CT26 mouse colorectal carcinoma cell culture, AaLS/LOX not only effectively consumed lactate but also generated a large amount of H_2_O_2_, which damaged cells and eventually induced necrotic cell death under normoxic conditions. In contrast, CAT of AaLS/LOX/CAT effectively converted highly toxic H_2_O_2_ to oxygen and water, preventing cell damage and death even with complete lactate consumption. Moreover, under the hypoxic culture condition, similar to the typical TME, while larger amounts of AaLS/LOX and AaLS/LOX/CAT were required to completely consume lactate, AaLS/LOX produced large amounts of H_2_O_2_ to kill CT26 cells, as in normoxia. Interestingly, unlike normoxic conditions, the AaLS/LOX/CAT treatment of CT26 cells under the hypoxic condition resulted in reduced cell viability, the lowest cell viability was observed around 100 nM of AaLS/LOX/CAT, where relatively small amounts of H_2_O_2_ was generated.

CT26 tumor-bearing allografted mice were subsequently established to evaluate the efficacy of AaLS/LOX/CAT in vivo and the local treatments of tumor-bearing mice with appropriate AaLS/LOX/CAT (10 μM) amounts effectively consumed lactate to modulate the TME and produced adequate amounts of H_2_O_2_, which was high enough to suppress tumor growth, but not too high to result in any serious side effects. Effective lactate consumption in the CT26 TME resulted in immune cell population alterations. Notably, AaLS/LOX/CAT treatments not only significantly reduced the population of tumor-supportive M2-like TAMs in tumors but also increased the population of tumor-suppressive neutrophils. Nevertheless, the combined effects of producing adequate H_2_O_2_ and altered tumor infiltrated immune cell species, driven by effective lactate consumption with AaLS/LOX/CAT, could efficiently suppress CT26 tumor growth in vivo. Therefore, the approach described here would provide opportunities that enhance the development of new enzyme complex-based therapeutic tools, suppressing tumors by controlling cancer metabolites and modulating the TME.

## Methods

### Chemicals

Sodium pyruvate, 5-(and-6)-chloromethyl-2,7,dichlorodihydrofluorescein diacetate, acetyl ester (CM-H_2_DCFDA), annexin V-FITC, and 7-Aminoactinomycin D (7-AAD) were obtained from Thermo Fisher Scientific (Waltham, MA, USA). N-Acetyl-l-cysteine (NAC) was purchased from Sigma-Aldrich (St. Louis, MO, USA). Mn (III) tetrakis-(4-benzoic acid)-porphyrin chloride (MnTBAP) and copper bis-3,5-diisopropylsalicylate (CuDIPs) were purchased from Abcam (Cambridge, UK).

### Molecular cloning, protein expression, and purification

LOX, CAT, and CBD genes were acquired through the gene synthesis (Bioneer, Daejeon, Korea). LOX originated from *Aerococcus viridans* [53] and CAT originated from Bacillus subtilis strain 168 (UniProt P26901) were utilized. CBD was originated from the A3 domain of von Willebrand factor (VWF) [54]. SC-LOX, SC-CAT, AaLS-ST, and ST-CBD genes were cloned into pETDuet-1 vector (Amp^R^) and recombinant DNAs were transformed into competent *E. coli* strain BL21 (DE3) (Additional file 1: Fig. S1). For the expression of each protein, cells were first grown in 1.0 L of Luria-Burtani (LB) medium containing 50 mg/L of an ampicillin at 37 °C until the optical density of 0.5–0.6. SC-LOX was induced with 50 μM IPTG and cultured overnight at 25 °C, and other proteins were induced with 500 μM IPTG and cultured overnight at 30 °C. The cells were harvested, lysed with lysozyme followed by sonication, and cellular debris was removed by centrifugation (15,000×*g*, 1 h, 4 °C). AaLS-ST subunits self-assemble to form an icosahedral protein cage nanoparticle structure spontaneously. The AaLS-ST purification process required additional heating (55 °C, 10 min) and centrifugation (15,000×*g*, 30 min, 4 °C). Proteins in the cell lysate were purified by fast protein liquid chromatography (FPLC, BioRad, Hercules, CA, USA) using immobilized metal affinity chromatography (IMAC, Nickel-nitrilotriacetic acid (Ni–NTA) agarose column). The IMAC-purified SC-LOX was concentrated by the centrifugal filters (Centricon, Merck-Millipore, Darmstardt, Germany) and further purified with size exclusion chromatography (SEC, superose 6 column, Cytiva, USA) using FPLC to remove any residual impurities. The endotoxin was removed by using Triton X-114 (Sigma, USA), and the levels of the resulting samples were quantified by using a Limulus Amebocyte Lysate assay (GenScript, Netherlands). The resulting levels were all less than 0.15 EU/mL [55].

### Characterization of AaLS variants

For constructing AaLS/LOX, approximately 40% of AaLS-ST subunits were ligated with SC-LOX (mol %). For AaLS/LOX/CAT, approximately 50% of SC-CAT tetramers were first pre-blocked with the ST-fused small collagen-binding domain (ST-CBD) and equivalent amounts of CBD-passivated SC-CAT and SC-LOX were subsequently ligated to AaLS-ST remaining approximately 20% of AaLS-ST subunits unmodified. Hydrodynamic diameters and zeta potentials of AaLS variants were measured using dynamic light scattering (DLS, Zetasizer, Malvern Panalytical, UK) with a disposable rectangular polystyrene cuvette. Each sample solution was prepared in phosphate buffer (pH 7.4, 50 mM Na_2_PO_4_, 100 mM NaCl) and adjusted to 25 °C before introduction to the instrument. The system was operated at 25 °C, equilibrated for 2 min, and scattered light was measured at 90° angle with the projected light. The samples were further analyzed with size exclusion chromatography (SEC, Superose 6 column, Cytiva, USA). The system was operated at a flow rate of 0.5 mg/min with FPLC. AaLS variants (AaLS-ST, AaLS/LOX, and AaLS/LOX/CAT) maintained their structure and there was no sign of aggregation or degradation even after being kept at 4 °C for more than a month.

TEM experiments were carried out on JEOL-1400 Bio-TEM operated at an acceleration voltage of 120 kV. TEM samples were prepared by placing 10 μL of the samples on carbon-coated copper grids (Electron Microscopy Sciences, Hatfield, PA, USA). The samples were incubated on the grid for 1 min and residual solutions were removed with a filter paper. The samples were negatively stained by applying 5 μL uranyl acetate (1% w/v) onto the grid and incubating for 1 min. The excess uranyl acetate solution was removed with a filter paper and the samples were left to dry overnight before imaging.

### Enzyme activity measurements

The LOX activity was determined by measuring amounts of H_2_O_2_ produced using the coupling reaction with horseradish peroxidase (HRP) and Amplex Red (Thermo Fisher Scientific, Waltham, MA, USA) by using the plate reader (530 nm absorbance, SpectraMAX 190, Molecular Devices, San Jose, CA, USA) in a real-time. The CAT activity was determined by monitoring colorimetric changes at 410 nm with the plate reader upon the addition of ammonium molybdate, which reacts with residual H_2_O_2_, when the CAT reaction was stopped. Initial velocity of each concentration was plotted and plots were fitted with the Michaelis-Menten equation for the calculation of *K*_cat_ and *K*_m_ values.

### Lactate consumption measurements in the buffer

The reaction buffer was prepared by adding 10 mM lactate to the PBS and pH was adjusted to 6.7. Each reaction buffer was treated with 100 nM of samples. For normal condition, the buffer was loaded to the 96-well plate and incubated in the 37 °C humidified incubator with samples. To create the hypoxic condition, the buffer was loaded to the glass vial and dissolved oxygen was removed by bubbling with N_2_ gas. The glass vial was subsequently blocked with rubber septa and sealed with a crimper. At each time point, samples were collected with the syringe, the reactions were directly inactivated with metaphosphoric acid (MPA), and the lactate concentrations were measured with a l-lactate assay kit (Cayman Chemical, Ann Arbor, MI, USA).

### Cell viability and lactate assays

CT26 cells were cultured in RPMI-1640 supplemented with 10% FBS and 1% antibiotic–antimycotic. The cells were cultured in a humidified atmosphere of 5% CO_2_, 37 °C with 95% air (normoxic culture) or 1% O_2_ (hypoxic culture). Cells (1 × 10^4^ per well, 100 μL) were seeded in a flat-bottomed 96-well plate (white, clear bottom, Greiner, Austria) and incubated at 37 °C in 5% CO_2_ for 24 h. Depending on the culture condition, the cells were either kept in the normoxic condition or transferred to the hypoxic chamber (37 °C, 1% O_2_ and 5% CO_2_) and cultured for 24 h. 20 μL of samples were added to corresponding wells and incubated for additional 24 h. After 72 total culture hours, 20 μL of sample-treated cell culture media were collected from each well and directly inactivated with metaphosphoric acid (MPA) for the measurement of lactate concentration using a l-lactate assay kit (Cayman Chemical, Ann Arbor, MI, USA). The remaining cells in 96-well plates were treated with CellTiter-Glo solution (Promega, Madison, WI, USA) and incubated for 10 min on the orbital shaker. The luminescence intensities of each well were recorded with the plate luminescence reader (Tecan Infinite M200 pro, Switzerland) and the cell viability of each well were determined. All experiments were performed in triplicates.

### Flow cytometry

CT26 cells were grown on 6-well plates for 48 h and treated with 4 nM of AaLS-ST, AaLS/LOX, or AaLS/LOX/CAT for 6 or 12 h. Positive control was prepared by incubating cells at 65 °C for 5 min. Cells were harvested with trypsin, washed with ice-cold PBS, and stained with annexin V-FITC and 7-AAD (Thermo Fisher Scientific, Waltham, MA, USA) for 15 min at 37 °C according to the manufacturer’s instructions. The stained cells were analyzed by NovoCyte 3000 Flow Cytometer (Agilent, Santa Clara, CA, USA).

### Live-cell imaging

CT26 cells were allowed to grow for 48 h before the addition of AaLS-ST, AaLS/LOX, or AaLS/LOX/CAT (4 nM), and subsequently monitored for 12 h in a humidified live cell incubating system (Live Cell Instrument, Stage-top Incubator System T, Gyeonggi-do, Republic of Korea) with 5% CO_2_ atmosphere and 37 °C. Images were acquired at 20-min intervals using fluorescence microscopy (Carl Zeiss, AxioObserver 7, Oberkochen, Germany).

### ROS detection

ROS production was visualized using the dye CM-H_2_DCFDA (Thermo Fisher Scientific, Waltham, MA, USA) by fluorescence microscopy (Carl Zeiss, AxioObserver 7, Oberkochen, Germany). Cells treated with AaLS-ST, AaLS/LOX, or AaLS/LOX/CAT were incubated with 2 μM of CM-H2DCFDA for 30 min in the dark at 37 °C. After washing with Hank’s Buffered Salt Solution (HBSS) containing calcium and magnesium, cells were further observed by fluorescence microscopy.

### In vivo experiments

For the tumor-bearing mouse model, 2 × 10^6^ cells of CT26 cells were injected at the right flank of the female balb/c mice. After the volume of the tumors reached approximately 100 mm^3^, PBS, AaLS, AaLS/LOX, or AaLS/LOX/CAT were intratumorally administrated for six times in two- or three-day intervals and the tumor sizes and body weights were monitored (n = 4 per group). The tumor size was assessed by periodic caliper measurements and tumor volume was calculated using a formula, (length × width^2^)/2. The mice were sacrificed at 15 or 16 days after injections and the tumor masses and organs were collected by biopsy at the last day for the further immune profiling and H&E staining. Images of H&E staining tissue sections were captures and processed using an Olympus virtual microscope (BX51/dotSlide, Olympus, Japan). All in vivo experiments were performed under the guidance of the Institutional Animal Care and Use Committee of Ulsan National Institute of Science and Technology (UNISTIACUC, UNISTIACUC-21-31) in this study.

### Immune profiling

Mice were anesthetized, and mouse tumors were harvested. Whole tumors were minced into the small pieces, followed by digestion in collagenase IA (350 μg/ml: C9891, Sigma-Aldrich), hyaluronidase (100 μg/mL: H3506, Sigma-Aldrich) and DNase I (250 μg/ml: 10104159001, Roche) in RPMI 1640 containing FBS (5%) at 37 °C with shaking at 160 rpm for 30 min. The digested tissue was filtered through 70-μm nylon cell strainer and red blood cells were lysed using ACK lysis buffer (A1049201, Roche). Cells were washed resuspended in PBS (containing 2% FBS) and pelleted at 1500 rpm for 5 min. To isolate CD45-positive tumor infiltrating leukocytes (TILs), cells were resuspended with MACS buffer and separated with MACS column using CD45 (TIL) MicroBeads (130-11-618, Miltenyi Biotec). Isolated CD45-positive TILs were counted, and live cells were identified by Zombie Aqua™ Fixable Viability Kit (413202, BioLegend). Then, cells were incubated with TruStain FcX (101319, BioLegend) before staining with specific cell surface antibodies. The following antibodies were used for flow cytometry analysis: CD45-BV421 (103133, BioLegend), Ly6G-BV650 (127641, BioLegend), CD4-PE (100407, BioLegend), CD8a-FITC (100705, BioLegend), CD11b-PE-cy7 (101215, Biolegend), CD64-APC (139305, BioLegend), CD3-PerCP-cy5.5 (100217, BioLegend), NK1.1-APC-cy7 (108723, BioLegend), F4/80-FITC (123108, BioLegend), F4/80-APC (17-4801-82, eBioscience), CD206-PE (141706, BioLegend), and CD86-PE (159204, BioLegend). Data were acquired on LSR Fortessa cell analyzer (BD Biosciences) and analyzed using FlowJo Software.

### Isolation and culture of mouse BMDM.

To obtain the mouse BMDM, proximal and distal ends of the femurs and tibias were transversally cut and the bone marrow was flushed out by injecting 5 mL of Corning Dulbecco’s Modified Eagle Medium (DMEM) supplemented with 10% heat-inactivated FBS, 5% pen-strep, and 5% l-glutamine into the medullary canal with a syringe and 25-gauge needle. Cells were centrifuged and resuspended in DMEM and further supplemented with mouse macrophage colony-stimulating factor (10 ng/mL; Peprotech). Cells were plated into four 10 cm diameter cell culture dishes and cultured for 5 days at 37 °C and 5% CO_2_. After reaching confluence, cells were lifted with gentle scraping, centrifuged, and split into six-well culture dishes and stimulated with lactate (25 mM) in the presence of AaLS or AaLS/LOX/CAT.

### Statistical analysis

All statistical analysis was performed using GraphPad Prism 7. All data are represented as the mean ± SD (standard deviation) of at least three separate experiments, as described in individual figure legends. Statistical differences were determined using the one-way or two-way analysis of variance (ANOVA) followed by Dunnett’s or Turkey’s post hoc test for multiple comparisons. *p*-values less than 0.05 were considered statistically significant.

## Supplementary Information

**Additional file 1: Figure S1.** Amino acid sequence information. SpyTag and SpyCatcher amino acid sequences are indicated as red and blue colors, respectively. **Figure S2.** Characterization of SC-LOX and SC-CAT and their ligated products with AaLS-ST. (a) SDS-PAGE analyses of purified SC-LOX and SC-CAT. (b) Size exclusion chromatography elution profiles of LOX, SC-LOX, CAT, SC-CAT, and M/W standard protein mixture solution. (c) Estimation of the molecular weight of purified proteins compared with M/W standard protein mixture elution times. Estimated molecular weights of each protein and size exclusion elution time are listed. SDS-PAGE analyses of the ligation reaction resultants of AaLS-ST and SC-LOX (d) or AaLS-ST and SC-CAT (e). The final lane of each gel is the supernatant of the centrifuged sample solution (13,000 × g, 10 min, 4 °C) representing leftovers after protein aggregation. (f) Schematic illustration of SC-CAT immobilization to the surface of AaLS-ST after partial passivation with ST-CBD to avoid protein aggregation. (g) SDS-PAGE analyses of the ligation reaction resultants of SC-CAT (red arrow) with various amounts of ST-CBD (blue arrow). (h) SDS-PAGE analyses of the ligation reaction resultants of AaLS-ST with partially CBD-passivated SC-CAT before and after centrifugation. The degree of protein aggregation is estimated by determining the amounts of loss after centrifugation. (i) SDS-PAGE analyses of the ligation resultants of AaLS-ST (black arrow) with SC-LOX (yellow arrow) and CBD-passivated SC-CAT (red and blue arrows) before and after centrifugation. The degree of protein aggregation is estimated by determining the amounts of loss after centrifugation. (j) Zeta potential measurements of AaLS-ST, AaLS/LOX, and AaLS/LOX/CAT. **Figure S3.** Surface and ribbon diagram representations and transmission electron microscopic images of LOX and CAT. (a) Surface and ribbon diagram representations of LOX (PDB ID: 2DU2) are shown as top and side views. The N-terminal four residues are shown as spheres in red. All four N-termini face one direction. (b) Surface and ribbon diagram representations of homologous CAT (PDB ID: 1SI8) are shown as top and side views. The N-terminal four residues are shown as spheres in blue. Two N-termini each face opposite directions. (c) Transmission electron microscopic images of SC-LOX, SC-CAT, and AaLS-ST stained with 1% uranyl acetate. Size bars are included as indicated. **Figure S4.** (a) Lactate concentrations at various incubation times of CT26 cells under the normoxic culture condition. (b) Microscopic images of CT26 cells treated with various amounts of AaLS-ST, AaLS/LOX, or AaLS/LOX/CAT under the normoxic condition. (c) Cell viability of CT26 cells subjected to different treatments under the normoxic condition. CT26 cells were pretreated with the indicated concentrations of NAC, sodium pyruvate, CuDIPs, or MnTBAP and were then treated with 4 nM AaLS-ST, AaLS/LOX, or AaLS/LOX/CAT for 24 h. Cell viability was measured using CellTiter-Glo Luminescent Cell Viability Assay. Data are represented as means ± SD, n = 3, *p < 0.05; **p < 0.01; ***p < 0.001; ****p < 0.0001. **Figure S5.** Detection of ROS generation induced by AaLS/LOX and AaLS/LOX/CAT under the hypoxic condition. CT26 cells were treated with AaLS-ST, AaLS/LOX, or AaLS/LOX/CAT for indicated times and were then incubated with CM-H_2_DCFDA prior to fluorescence microscopy (scale bar, 100 μm). **Figure S6.** In vivo therapeutic efficacy of AaLS/LOX and AaLS/LOX/CAT. (a) Mice survivals treated with PBS, AaLS/LOX/CAT (5 or 10 μM), or AaLS/LOX (5 or 10 μM). (b) Body weights were measured in mice treated with PBS or AaLS/LOX/CAT (5 or 10 μM) every 2 or 3 days. (c) Tumor sizes were measured in mice treated with PBS or AaLS/LOX/CAT (5 or 10 μM) every 2 or 3 days using a caliper. Data are averages ± standard deviations (SD); n = 3 per group; n.s. not significant. (d) Tumor sizes were measured in mice treated with PBS, or 10 or 20 μM AaLS/LOX/CAT every 2 or 3 days using a caliper. Data are averages ± standard deviations (SD); n = 3 per group; *p < 0.05. (e) Body weights were measured in mice treated with PBS, or 10 or 20 μM AaLS/LOX/CAT every 2 or 3 days. **Figure S7.** Immune profiling of tumors. (a) Gating strategies for flow cytometry analysis. Lymphocytes were defined as CD45^+^CD3^+^CD8^+^ T cells and CD45^+^CD3^+^CD4^+^ T cells, macrophages were CD45^+^CD64^+^, neutrophils were CD45^+^CD3^−^CD11b^+^Ly6G^+^, NK cells were CD45^+^CD3^−^CD11b^−^NK1.1^+^. (b) Representative FACS dot plots of lymphocytes (CD4^+^ and CD8^+^ T cells) and NK cells in tumors of indicated conditions. Graph on the right shows the quantification of the frequency of CD4^+^ T, CD8^+^ T, and NK cells (n = 3). *p < 0.05, ns, not significant, by student’s t-test. **Figure S8.** The effect of lactate upon the treatment with AaLS or AaLS/LOX/CAT on macrophage phenotype polarization. (a) The lactate concentrations in the media of CT26 cell cultures or treated with either AaLS or AaLS/LOX/CAT. (b) FACS plots showing CD206^+^ bone-marrow-derived macrophages (BMDM) treated with AaLS or AaLS/LOX/CAT in the presence of additional lactate (25 mM) in the indicated conditions. (c) Expression analysis by qPCR of Arg1 and Vegf mRNA in bone-marrow-derived macrophages (BMDM) treated with either AaLS or AaLS/LOX/CAT grown under the additional lactate (25 mM) in the indicated conditions.

## Data Availability

The datasets used during the current study are available from the corresponding author on reasonable request.
