# Supplementary material for: Lactate oxidase/catalase-displaying nanoparticles efficiently consume lactate in the tumor microenvironment to effectively suppress tumor growth
Source: J Nanobiotechnology. 2023 Jan 3;21:5. doi: 10.1186/s12951-022-01762-6 (PMC9811728; doi:10.1186/s12951-022-01762-6)
Supplement: Supplementary file 1 — Additional file 1: Figure S1. Amino acid sequence information. SpyTag and SpyCatcher amino acid sequences are indicated as red and blue colors, respectively. Figure S2. Characterization of SC-LOX and SC-CAT and their ligated products with AaLS-ST. (a) SDS-PAGE analyses of purified SC-LOX and SC-CAT. (b) Size exclusion chromatography elution profiles of LOX, SC-LOX, CAT, SC-CAT, and M/W standard protein mixture solution. (c) Estimation of the molecular weight of purified proteins compared with M/W standard protein mixture elution times. Estimated molecular weights of each protein and size exclusion elution time are listed. SDS-PAGE analyses of the ligation reaction resultants of AaLS-ST and SC-LOX (d) or AaLS-ST and SC-CAT (e). The final lane of each gel is the supernatant of the centrifuged sample solution (13,000 × g, 10 min, 4 °C) representing leftovers after protein aggregation. (f) Schematic illustration of SC-CAT immobilization to the surface of AaLS-ST after partial passivation with ST-CBD to avoid protein aggregation. (g) SDS-PAGE analyses of the ligation reaction resultants of SC-CAT (red arrow) with various amounts of ST-CBD (blue arrow). (h) SDS-PAGE analyses of the ligation reaction resultants of AaLS-ST with partially CBD-passivated SC-CAT before and after centrifugation. The degree of protein aggregation is estimated by determining the amounts of loss after centrifugation. (i) SDS-PAGE analyses of the ligation resultants of AaLS-ST (black arrow) with SC-LOX (yellow arrow) and CBD-passivated SC-CAT (red and blue arrows) before and after centrifugation. The degree of protein aggregation is estimated by determining the amounts of loss after centrifugation. (j) Zeta potential measurements of AaLS-ST, AaLS/LOX, and AaLS/LOX/CAT. Figure S3. Surface and ribbon diagram representations and transmission electron microscopic images of LOX and CAT. (a) Surface and ribbon diagram representations of LOX (PDB ID: 2DU2) are shown as top and side views. The N-terminal f [file 12951_2022_1762_MOESM1_ESM.docx]

*Additional file 1*

**Lactate Oxidase/Catalase-displaying Nanoparticles Efficiently Consume Lactate in the Tumor Microenvironment to Effectively Suppress Tumor Growth**

Hyukjun Choi,^#^ Mirae Yeo,^#^ Yujin Kang,^#^ Hyo Jeong Kim, Seong Guk Park, Eunjung Jang, Sung Ho Park,* Eunhee Kim,* and Sebyung Kang*

Department of Biological Sciences, Ulsan National Institute of Science and Technology (UNIST), Ulsan, 44919, Korea

^#^These authors contributed equally to this work

Corresponding authors

Sebyung Kang: [sabsab7@unist.ac.kr](mailto:sabsab7@unist.ac.kr)

Eunhee Kim: [ehkim@unist.ac.kr](mailto:ehkim@unist.ac.kr)

Sung Ho Park: [parksh@unist.ac.kr](mailto:parksh@unist.ac.kr)

Figure S1.

**AaLS-ST**

MGSSHHHHHHSQDPNSSSMQIYEGKLTAEGLRFGIVASRFNHALVDRLVEGAIDSIVRHGGREEDITLVRVPGSWEIPVAAGELARKEDIDAVIAIGVLIRGATPHFDYIASEVSKGLANLSLELCKPITFGVITADTLEQAIERAGTKHGNKGWEAALSAIEMANLFKSLRKDPNSGGGLVARGSGGGSGGGTGGGSGGGVDNKFNKEQQNAFYEILHLPNLNEEQRNAFIQSLKDDPSQSANLLAEAKKLNDAQAPKGSGGAHIVMVDAYKPTK

**SC-LOX**

MGSSHHHHHHSQDPMVDTLSGLSSEQGQSGDMTIEEDSATHIKFSKRDEDGKELAGATMELRDSSGKTISTWISDGQVKDFYLYPGKYTFVETAAPDGYEVATAITFTVNEQGQVTVNGKATKGDAHIGGGGSGGGGSTSNNNDIEYNAPSEIKYIDVVNTYDLEEEASKVVPHGGFNYIAGASGDEWTKRANDRAWKHKLLYPRLAQDVEAPDTSTEILGHKIKAPFIMAPIAAHGLAHTTKEAGTARAVSEFGTIMSISAYSGATFEEISEGLNGGPRWFQIYMAKDDQQNRDILDEAKSDGATAIILTADSTVSGNRDRDVKNKFVYPFGMPIVQRYLRGTAEGMSLNNIYGASKQKISPRDIEEIAAHSGLPVFVKGIQHPEDADMAIKAGASGIWVSNHGARQLYEAPGSFDTLPAIAERVNKRVPIVFDSGVRRGEHVAKALASGADVVALGRPVLFGLALGGWQGAYSVLDYFQKDLTRVMQLTGSQNVEDLKGLDLFDNPYGYEY

**SC-CAT**

MGSSHHHHHHSQDPMVDTLSGLSSEQGQSGDMTIEEDSATHIKFSKRDEDGKELAGATMELRDSSGKTISTWISDGQVKDFYLYPGKYTFVETAAPDGYEVATAITFTVNEQGQVTVNGKATKGDAHIGGGGSGGGGSTSMSSNKLTTSWGAPVGDNQNSMTAGSRGPTLIQDVHLLEKLAHFNRERVPERVVHAKGAGAHGYFEVTNDVTKYTKAAFLSEVGKRTPLFIRFSTVAGELGSADTVRDPRGFAVKFYTEEGNYDIVGNNTPVFFIRDAIKFPDFIHTQKRDPKTHLKNPTAVWDFWSLSPESLHQVTILMSDRGIPATLRHMHGFGSHTFKWTNAEGEGVWIKYHFKTEQGVKNLDVNTAAKIAGENPDYHTEDLFNAIENGDYPAWKLYVQIMPLEDANTYRFDPFDVTKVWSQKDYPLIEVGRMVLDRNPENYFAEVEQATFSPGTLVPGIDVSPDKMLQGRLFAYHDAHRYRVGANHQALPINRARNKVNNYQRDGQMRFDDNGGGSVYYEPNSFGGPKESPEDKQAAYPVQGIADSVSYDHYDHYTQAGDLYRLMSEDERTRLVENIVNAMKPVEKEEIKLRQIEHFYKADPEYGKRVAEGLGLPIKKDS

**ST-CBD**

MGSSHHHHHHSAHIVMVDAYKPTKTSGGGSGGGASTGGGSGGGSGGGSGGGKNSTSTGGGSGGGCSQPLDVILLLDGSSSFPASYFDEMKSFAKAFISKANIGPRLTQVSVLQYGSITTIDVPWNVVPEKAHLLSLVDVMQREGGPSQIGDALGFAVRYLTSEMHGARPGASKAVVILVTDVSVDSVDAAADAARSNRVTVFPIGIGDRYDAAQLRILAGPAGDSNVVKLQRIEDLPTMVTLGNSFLHKLCSGFVRI

Figure S1. Amino acid sequence information. SpyTag and SpyCatcher amino acid sequences are indicated as red and blue colors, respectively.

Figure S2.


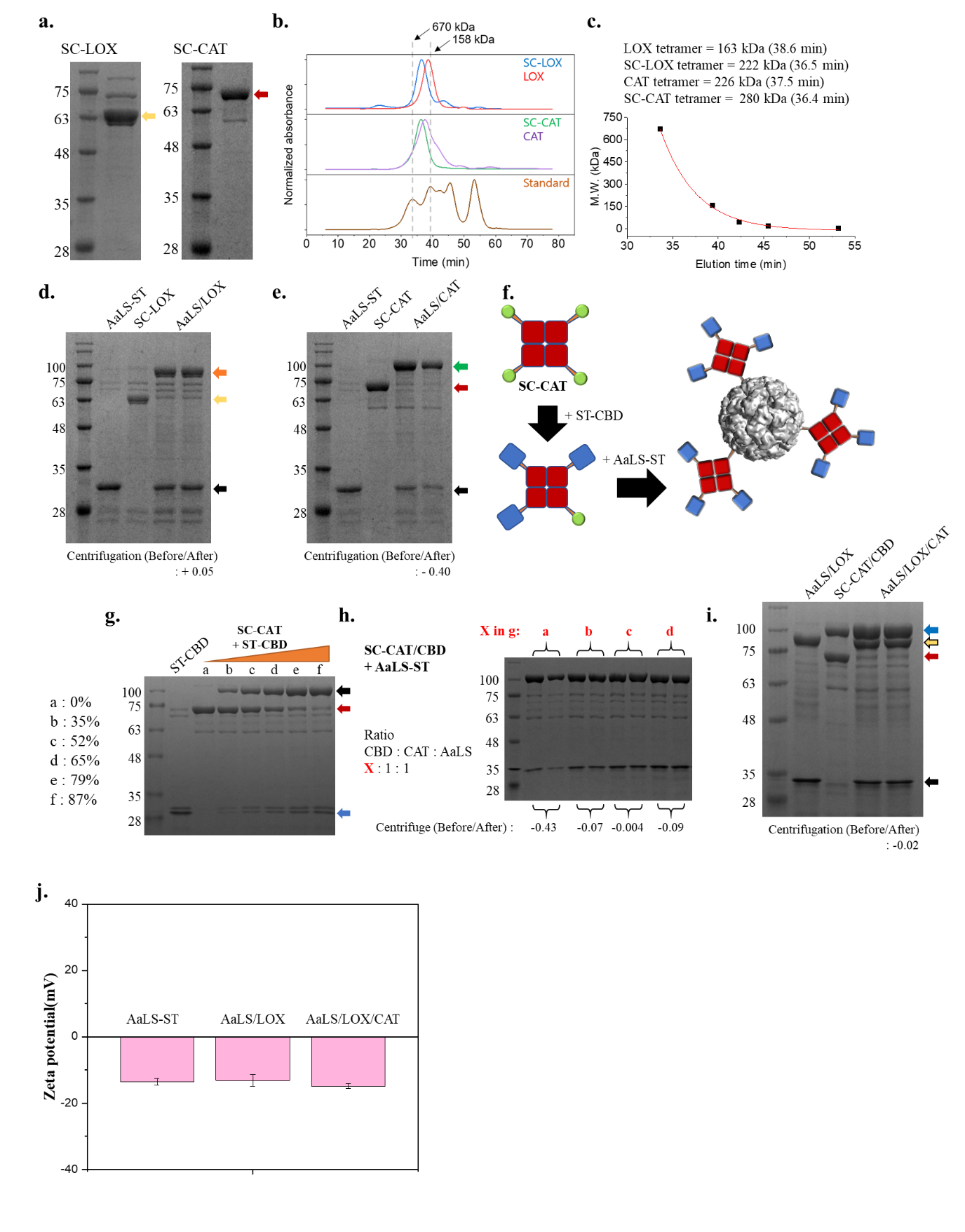


Figure S2. Characterization of SC-LOX and SC-CAT and their ligated products with AaLS-ST. (a) SDS-PAGE analyses of purified SC-LOX and SC-CAT. (b) Size exclusion chromatography elution profiles of LOX, SC-LOX, CAT, SC-CAT, and M/W standard protein mixture solution. (c) Estimation of the molecular weight of purified proteins compared with M/W standard protein mixture elution times. Estimated molecular weights of each protein and size exclusion elution time are listed. SDS-PAGE analyses of the ligation reaction resultants of AaLS-ST and SC-LOX (d) or AaLS-ST and SC-CAT (e). The final lane of each gel is the supernatant of the centrifuged sample solution (13,000 × g, 10 min, 4 °C) representing leftovers after protein aggregation. (f) Schematic illustration of SC-CAT immobilization to the surface of AaLS-ST after partial passivation with ST-CBD to avoid protein aggregation. (g) SDS-PAGE analyses of the ligation reaction resultants of SC-CAT (red arrow) with various amounts of ST-CBD (blue arrow). (h) SDS-PAGE analyses of the ligation reaction resultants of AaLS-ST with partially CBD-passivated SC-CAT before and after centrifugation. The degree of protein aggregation is estimated by determining the amounts of loss after centrifugation. (i) SDS-PAGE analyses of the ligation resultants of AaLS-ST (black arrow) with SC-LOX (yellow arrow) and CBD-passivated SC-CAT (red and blue arrows) before and after centrifugation. The degree of protein aggregation is estimated by determining the amounts of loss after centrifugation. (j) Zeta potential measurements of AaLS-ST, AaLS/LOX, and AaLS/LOX/CAT.


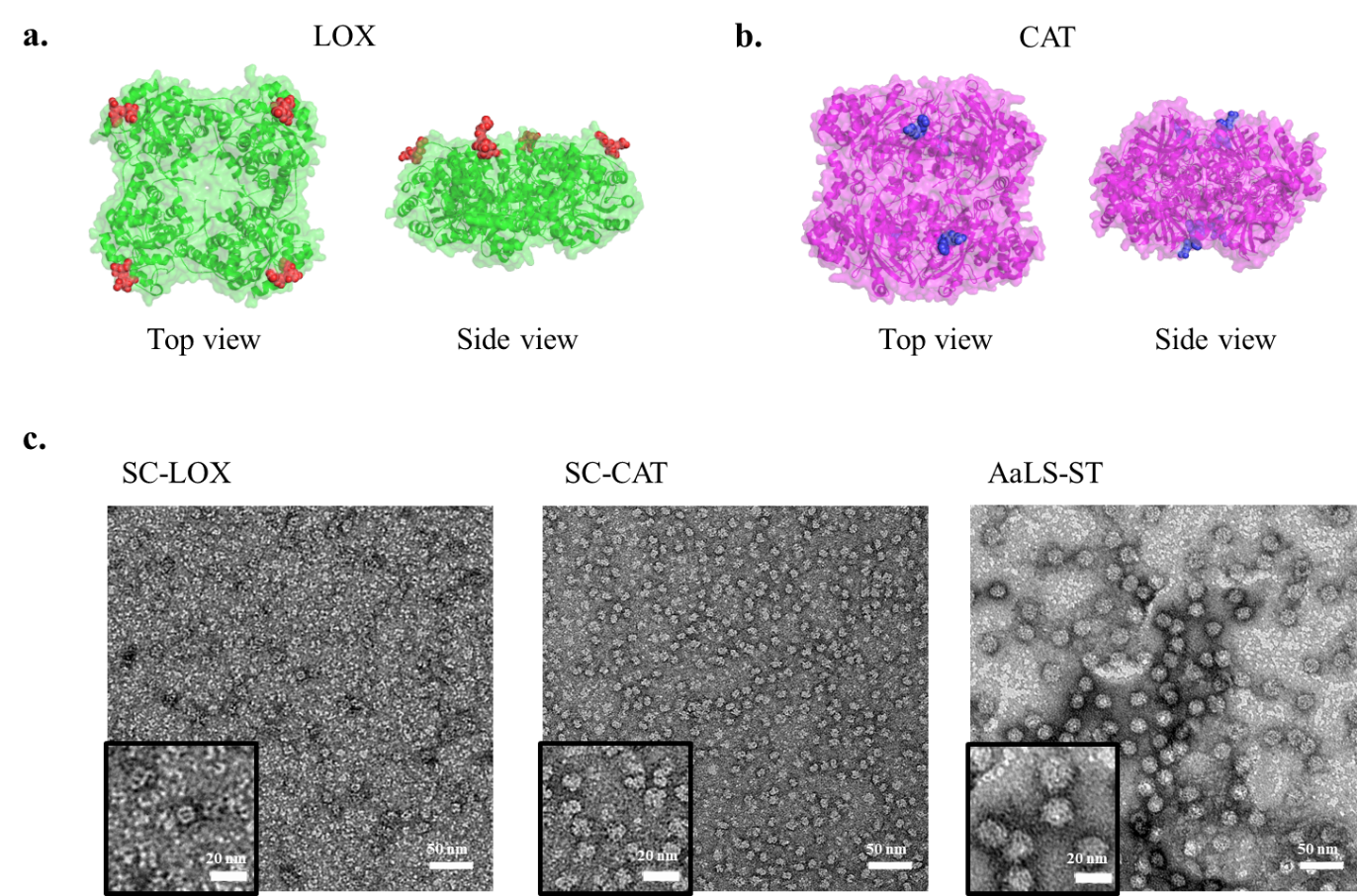
Figure S3.

Figure S3. Surface and ribbon diagram representations and transmission electron microscopic images of LOX and CAT. (a) Surface and ribbon diagram representations of LOX (PDB ID: 2DU2) are shown as top and side views. The N-terminal four residues are shown as spheres in red. All four N-termini face one direction. (b) Surface and ribbon diagram representations of homologous CAT (PDB ID: 1SI8) are shown as top and side views. The N-terminal four residues are shown as spheres in blue. Two N-termini each face opposite directions. (c) Transmission electron microscopic images of SC-LOX, SC-CAT, and AaLS-ST stained with 1% uranyl acetate. Size bars are included as indicated.


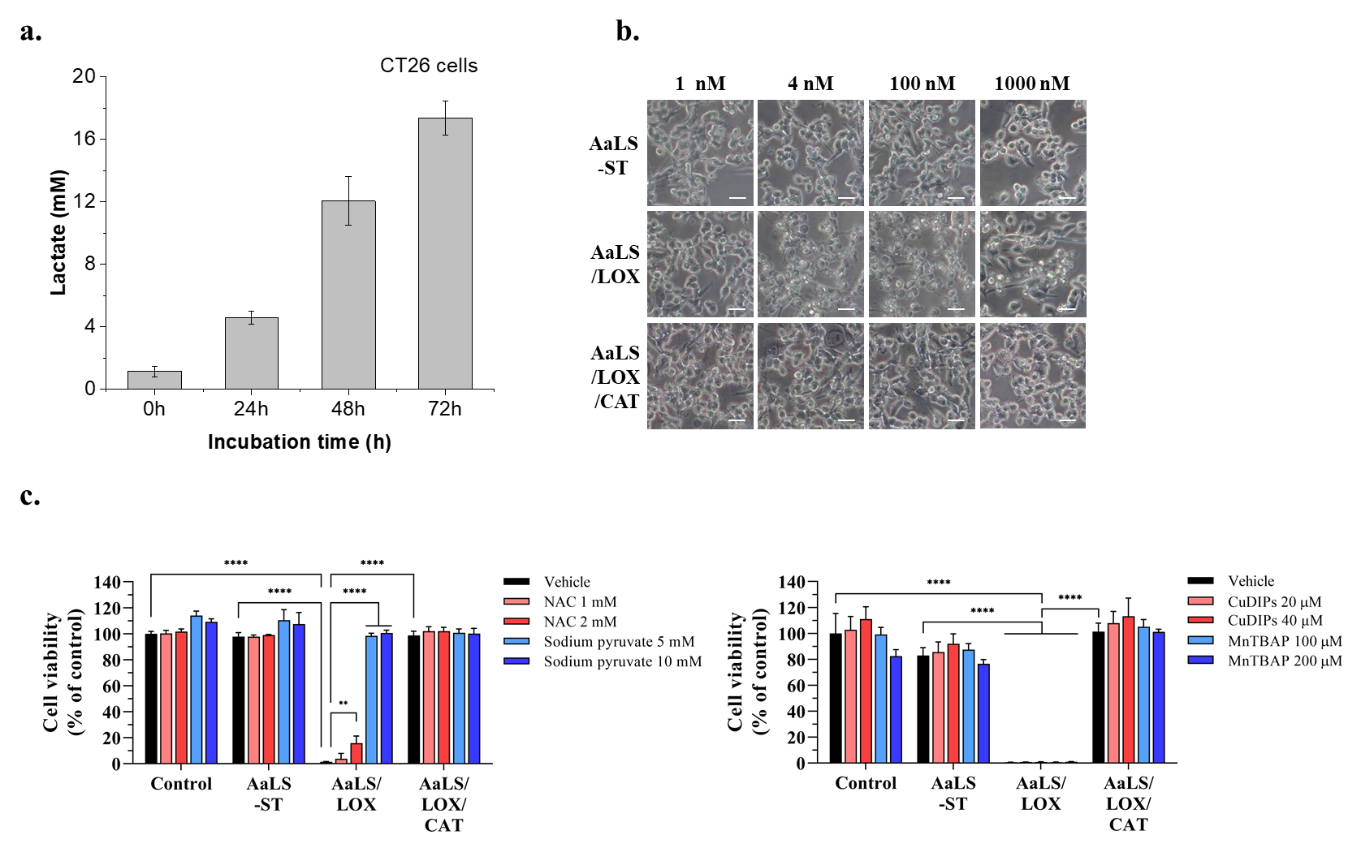
Figure S4.

Figure S4. (a) Lactate concentrations at various incubation times of CT26 cells under the normoxic culture condition. (b) Microscopic images of CT26 cells treated with various amounts of AaLS-ST, AaLS/LOX, or AaLS/LOX/CAT under the normoxic condition. (c) Cell viability of CT26 cells subjected to different treatments under the normoxic condition. CT26 cells were pretreated with the indicated concentrations of NAC, sodium pyruvate, CuDIPs, or MnTBAP and were then treated with 4 nM AaLS-ST, AaLS/LOX, or AaLS/LOX/CAT for 24 h. Cell viability was measured using CellTiter-Glo Luminescent Cell Viability Assay. Data are represented as means ± SD, n=3, **p* < 0.05; ***p* < 0.01; ****p* < 0.001; *****p* < 0.0001.


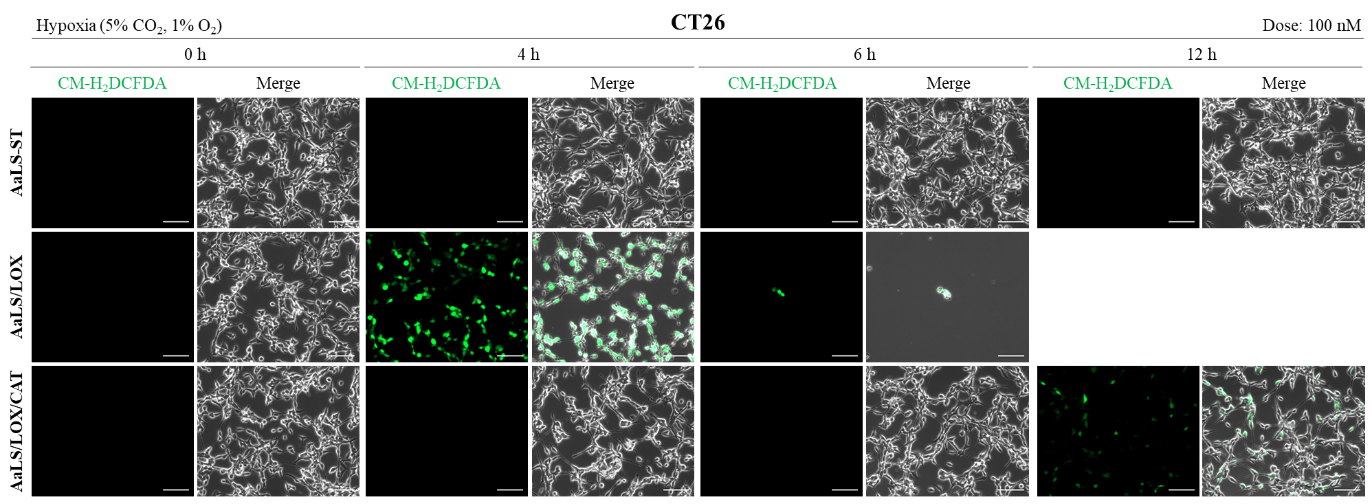
Figure S5.

Figure S5. Detection of ROS generation induced by AaLS/LOX and AaLS/LOX/CAT under the hypoxic condition. CT26 cells were treated with AaLS-ST, AaLS/LOX, or AaLS/LOX/CAT for indicated times and were then incubated with CM-H_2_DCFDA prior to fluorescence microscopy (scale bar, 100 μm).

Figure S6.


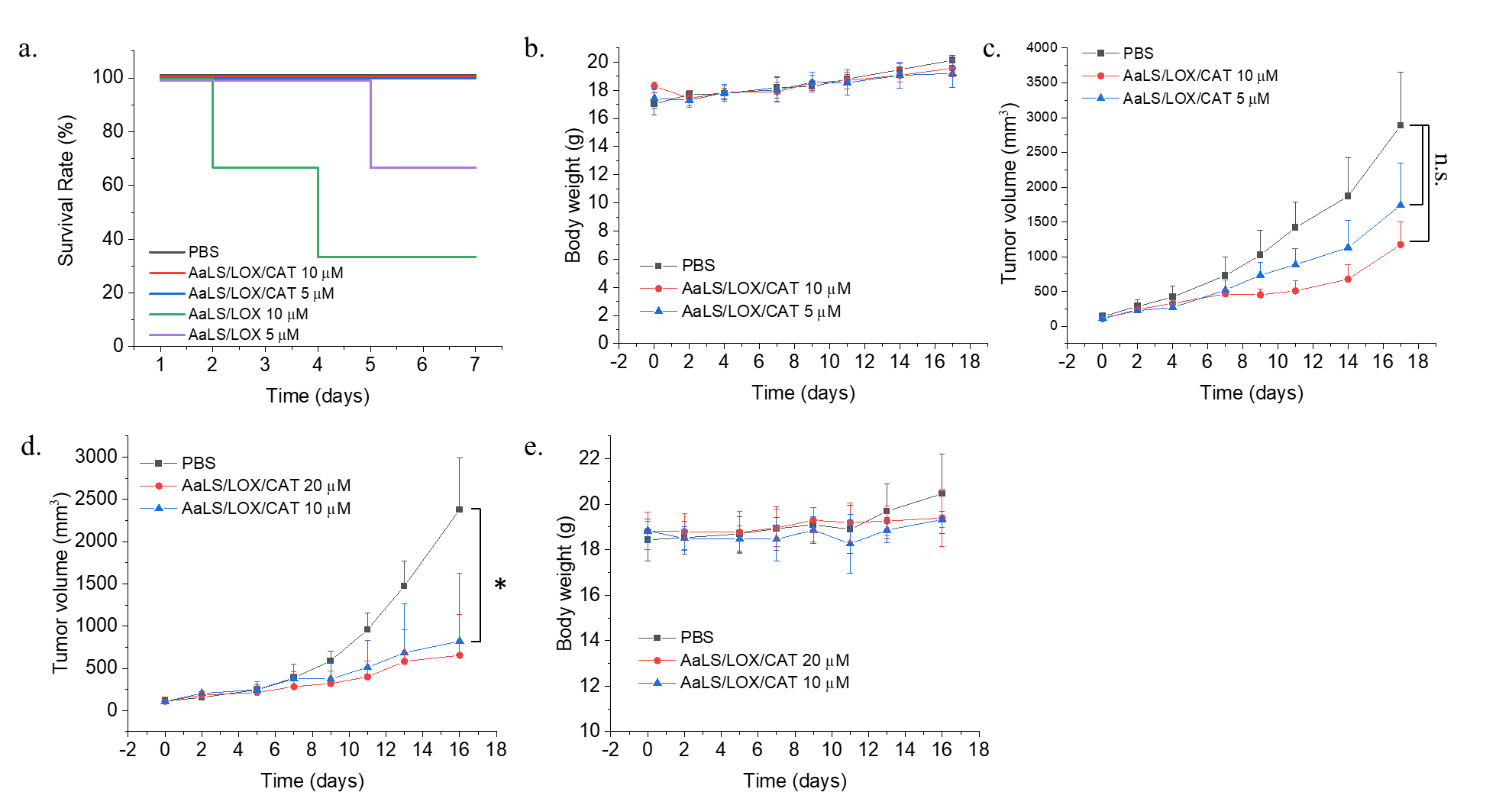


Figure S6. *In vivo* therapeutic efficacy of AaLS/LOX and AaLS/LOX/CAT. (a) Mice survivals treated with PBS, AaLS/LOX/CAT (5 or 10 μM), or AaLS/LOX (5 or 10 μM). (b) Body weights were measured in mice treated with PBS or AaLS/LOX/CAT (5 or 10 μM) every 2 or 3 days. (c) Tumor sizes were measured in mice treated with PBS or AaLS/LOX/CAT (5 or 10 μM) every 2 or 3 days using a caliper. Data are averages ± standard deviations (SD); n = 3 per group; n.s. not significant. (d) Tumor sizes were measured in mice treated with PBS, or 10 or 20 μM AaLS/LOX/CAT every 2 or 3 days using a caliper. Data are averages ± standard deviations (SD); n = 3 per group; **p* < 0.05. (e) Body weights were measured in mice treated with PBS, or 10 or 20 μM AaLS/LOX/CAT every 2 or 3 days.

Figure S7.


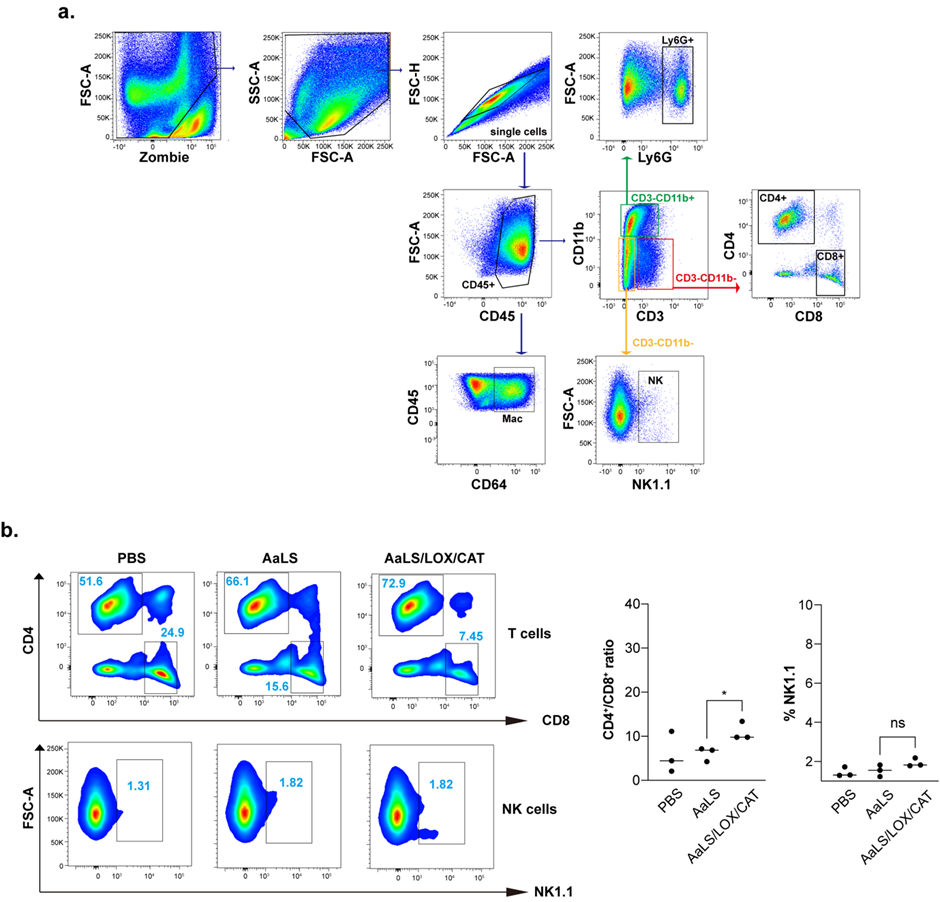


Figure S7. Immune profiling of tumors. (a) Gating strategies for flow cytometry analysis. Lymphocytes were defined as CD45^+^CD3^+^CD8^+^ T cells and CD45^+^CD3^+^CD4^+^ T cells, macrophages were CD45^+^CD64^+^, neutrophils were CD45^+^CD3^-^CD11b^+^Ly6G^+^, NK cells were CD45^+^CD3^-^CD11b^-^NK1.1^+^. (b) Representative FACS dot plots of lymphocytes (CD4^+^ and CD8^+^ T cells) and NK cells in tumors of indicated conditions. Graph on the right shows the quantification of the frequency of CD4^+^ T, CD8^+^ T, and NK cells (n = 3). *P<0.05, ns, not significant, by student’s t-test.

Figure S8.


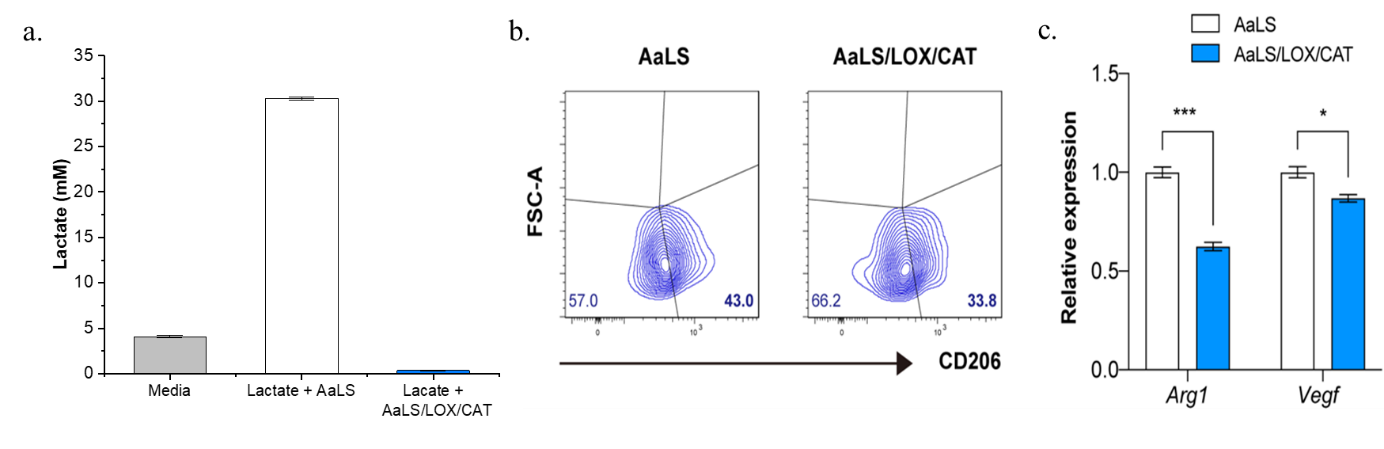


Figure S8. The effect of lactate upon the treatment with AaLS or AaLS/LOX/CAT on macrophage phenotype polarization. (a) The lactate concentrations in the media of CT26 cell cultures or treated with either AaLS or AaLS/LOX/CAT. (b) FACS plots showing CD206^+^ bone-marrow-derived macrophages (BMDM) treated with AaLS or AaLS/LOX/CAT in the presence of additional lactate (25 mM) in the indicated conditions. (c) Expression analysis by qPCR of Arg1 and Vegf mRNA in bone-marrow-derived macrophages (BMDM) treated with either AaLS or AaLS/LOX/CAT grown under the additional lactate (25 mM) in the indicated conditions.
